# Supplementary material for: Designed CXCR4 mimic acts as a soluble chemokine receptor that blocks atherogenic inflammation by agonist-specific targeting
Source: Nat Commun. 2020 Nov 25;11:5981. doi: 10.1038/s41467-020-19764-z (PMC7689490; doi:10.1038/s41467-020-19764-z)
Supplement: Supplementary file 1 — Supplementary Information [file 41467_2020_19764_MOESM1_ESM.pdf]

Supplementary Information

# Designed CXCR4 mimic acts as a soluble chemokine receptor that blocks atherogenic inflammation by agonist-specific targeting

Kontos et al.

This PDF file includes:
Supplementary Figures
Supplementary Tables

**Supplementary Figures**

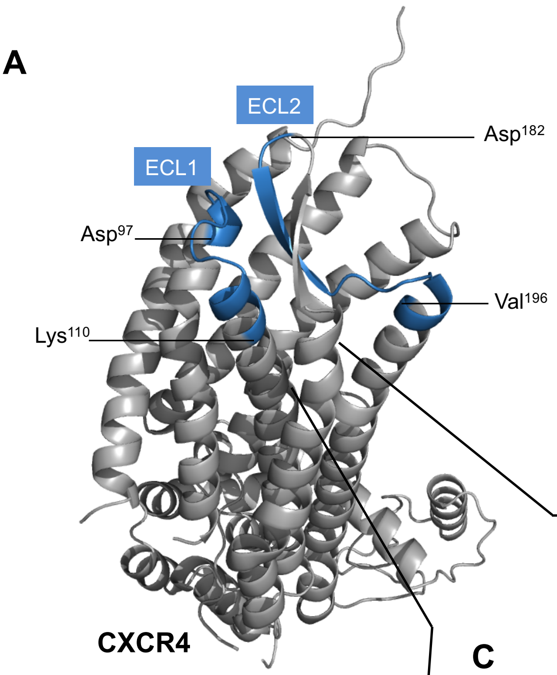


**B**

| Resolution (Å) | Distance between Lys110 and Asp182 (nm) | PDB code |
|---|---|---|
| 3.1 | 2.25 | 4RWS |
| 2.5 | 2.20 | 3ODU |
| 2.9 | 2.20 | 3OE0 |
| 3.2 | 2.22 | 3OE6 |
| 3.1 | 2.23 | 3OE8 |
| 3.1 | 2.18 | 3OE9 |

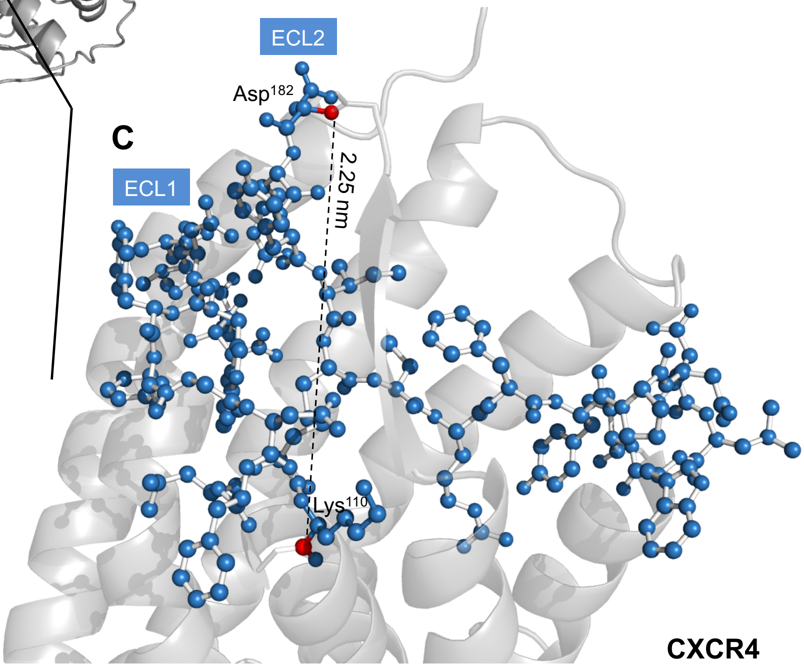


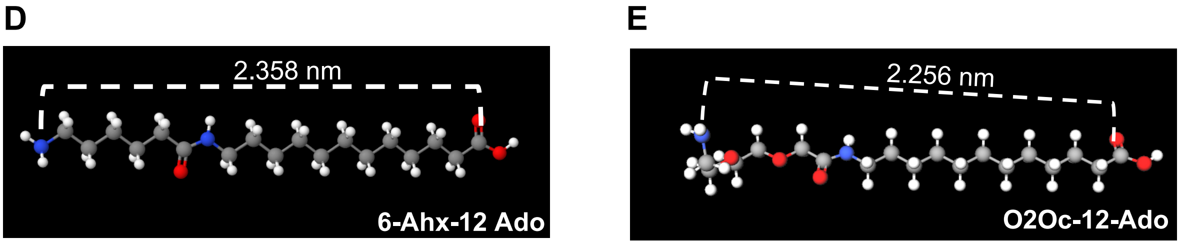


**Supplementary Figure 1** Structural basis for the design of the linker connecting ECL1 and ECL2 peptide sequences of human CXCR4. **a** Ribbon structure of human CXCR4 based on the crystal structure according to PDB code 4RWS [1]. Sequence stretches of extracellular loops ECL1 and ECL2 that were found to interact with MIF according to peptide array mapping [2] are highlighted in blue, and the N- and C-terminal residues of the ECL1 and 2 stretches are indicated. **b** Overview of distances between $Lys^{110}$ and $Asp^{182}$ of CXCR4 according to different crystal structure models[1, 3]. The respective resolutions of the structures and the PDB codes are indicated. **c** Zoomed top view of the CXCR4 ectodomain according to **a**; the measured length between the C-terminus of $Lys^{110}$ and the N-terminus of $Asp^{182}$ (2.25 nm) is shown. **d, e** Lengths of the 6-Ahx-12-Ado and O2Oc-12-Ado linkers as used in msR4M-L1 (**d**) and msR4M-L2 (**e**), respectively.

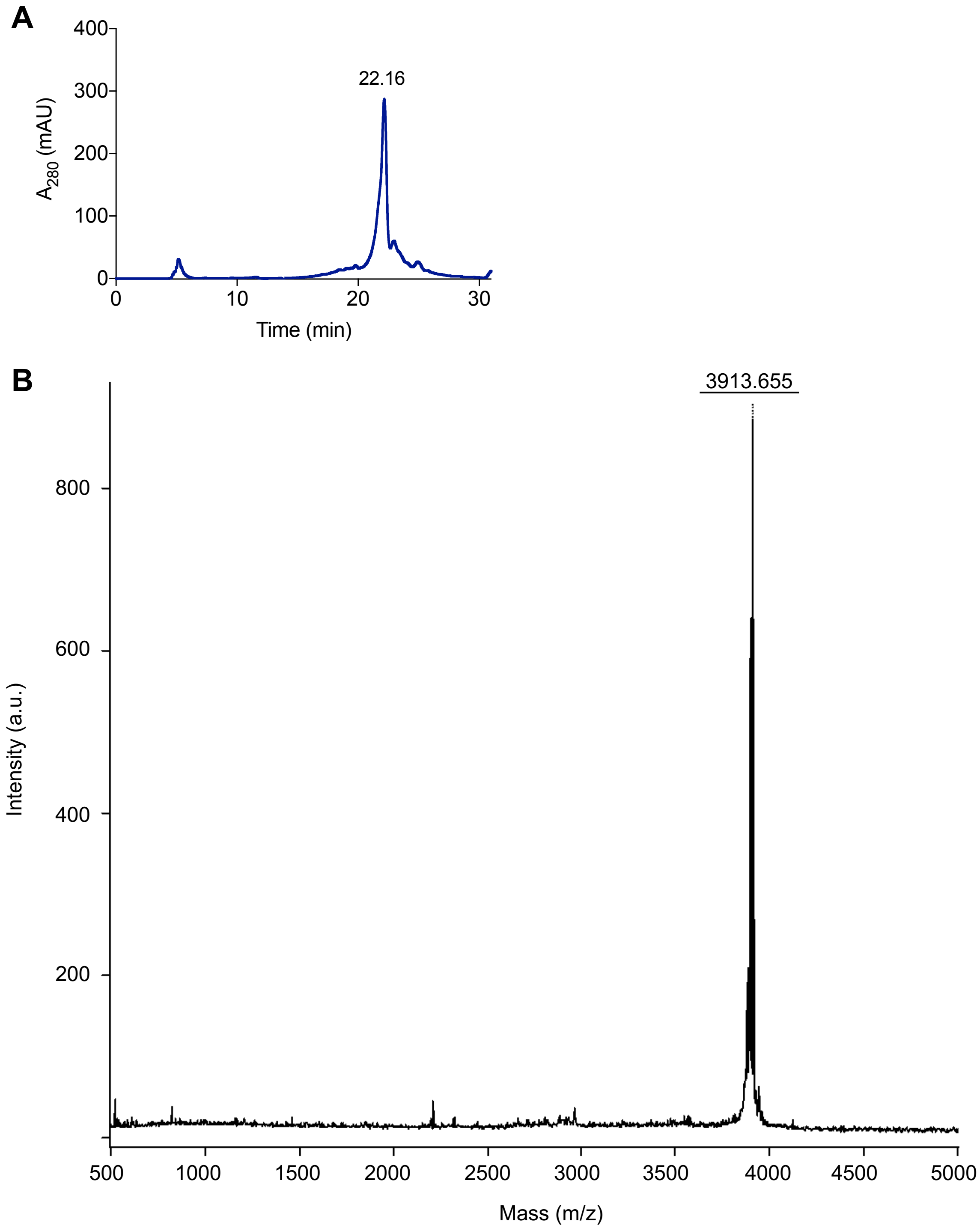


**Supplementary Figure 2** Purification of msR4M-L1 by HPLC and verification of peptide purity by mass spectrometric analysis. **a** Representative C18 HPLC chromatogram (absorbance 280 nm) of msR4M-L1 (retention time: 22.16 min; crude product) following solid phase peptide synthesis (SPPS). **b** Matrix-assisted laser desorption/ionization mass spectrometry (MALDI-MS) spectrum of HPLC-purified msR4M-L1. The theoretical calculated mass $[M+H]^+$ is 3912.92; the experimental mass $[M+H]^+$ is 3913.66.

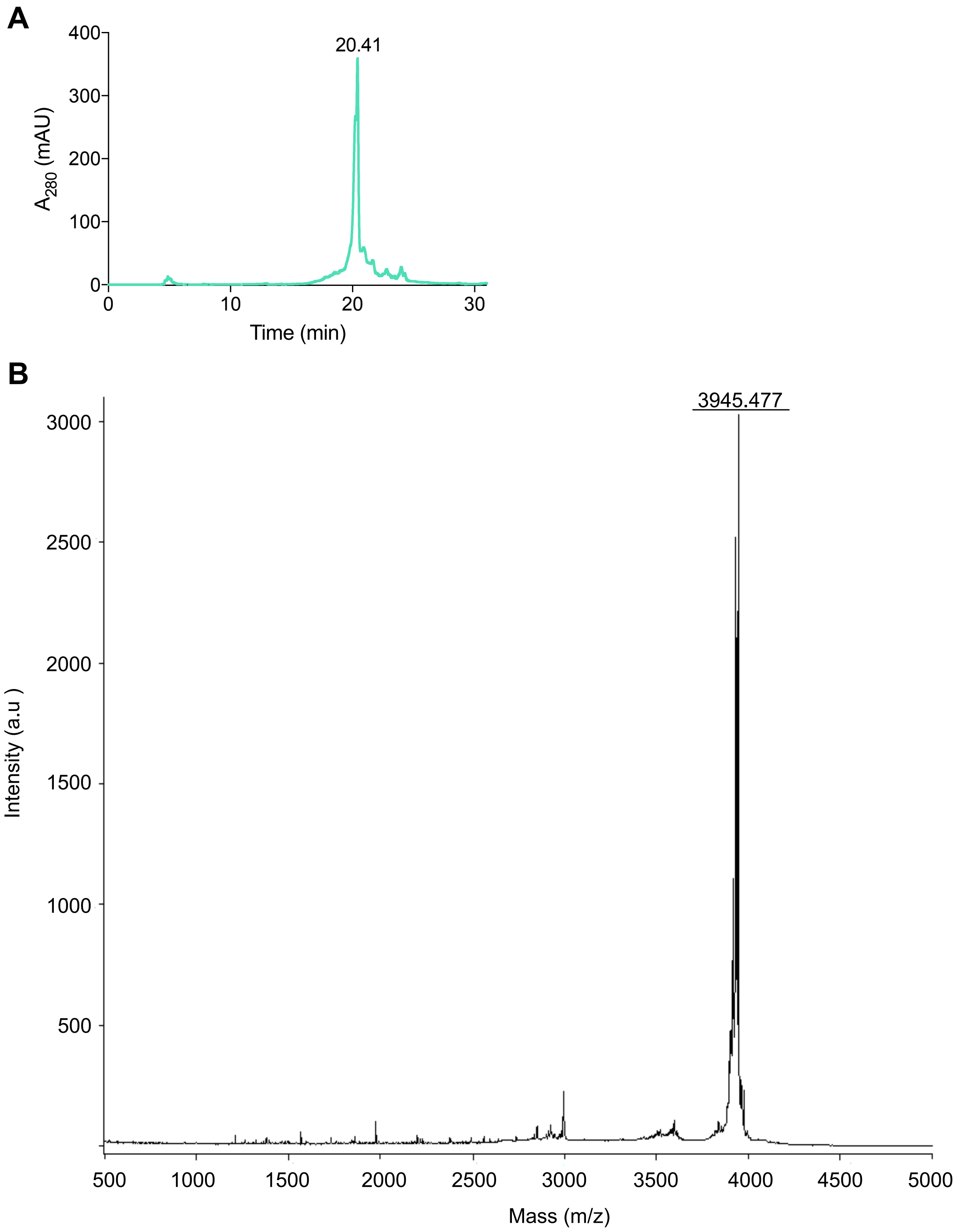


**Supplementary Figure 3** Purification of msR4M-L2 by HPLC and verification of peptide purity by mass spectrometric analysis. **a** Representative C18 HPLC chromatogram (absorbance 280 nm) of msR4M-L2 (retention time: 20.41 min; crude product) following solid phase peptide synthesis (SPPS). **b** Matrix-assisted laser desorption/ionization mass spectrometry (MALDI-MS) spectrum of HPLC-purified msR4M-L2. The theoretical calculated mass $[M+H]^+$ is 3944.93; the experimental mass $[M+H]^+$ is 3945.48.

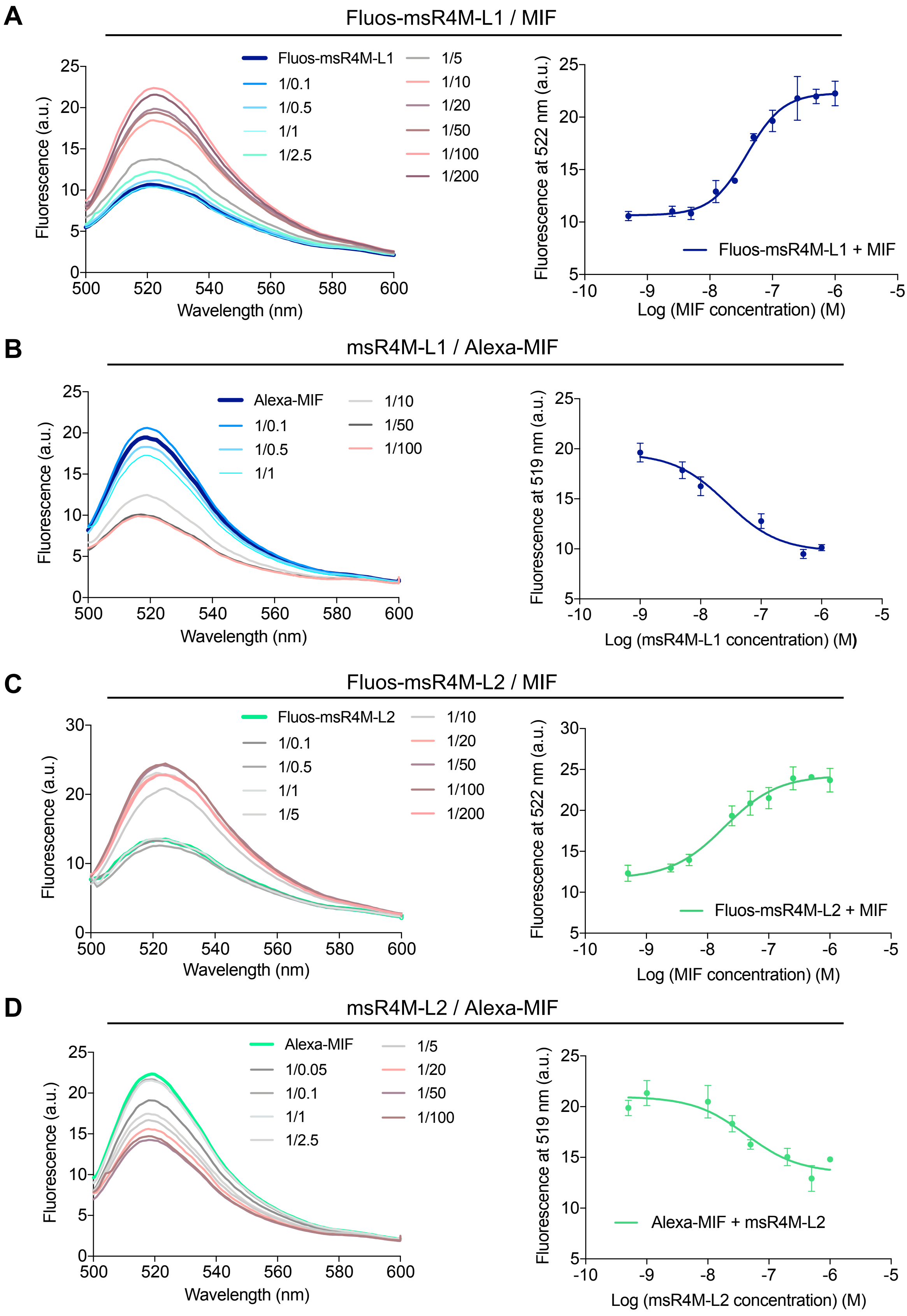
A
Fluos-msR4M-L1 / MIF
Fluorescence (a.u.)
Wavelength (nm)
Fluos-msR4M-L1
1/0.1
1/0.5
1/1
1/2.5
1/5
1/10
1/20
1/50
1/100
1/200
Fluorescence at 522 nm (a.u.)
Log (MIF concentration) (M)
Fluos-msR4M-L1 + MIF
B
msR4M-L1 / Alexa-MIF
Fluorescence (a.u.)
Wavelength (nm)
Alexa-MIF
1/0.1
1/0.5
1/1
1/10
1/50
1/100
Fluorescence at 519 nm (a.u.)
Log (msR4M-L1 concentration) (M)
C
Fluos-msR4M-L2 / MIF
Fluorescence (a.u.)
Wavelength (nm)
Fluos-msR4M-L2
1/0.1
1/0.5
1/1
1/5
1/10
1/20
1/50
1/100
1/200
Fluorescence at 522 nm (a.u.)
Log (MIF concentration) (M)
Fluos-msR4M-L2 + MIF
D
msR4M-L2 / Alexa-MIF
Fluorescence (a.u.)
Wavelength (nm)
Alexa-MIF
1/0.05
1/0.1
1/1
1/2.5
1/5
1/20
1/50
1/100
Fluorescence at 519 nm (a.u.)
Log (msR4M-L2 concentration) (M)
Alexa-MIF + msR4M-L2

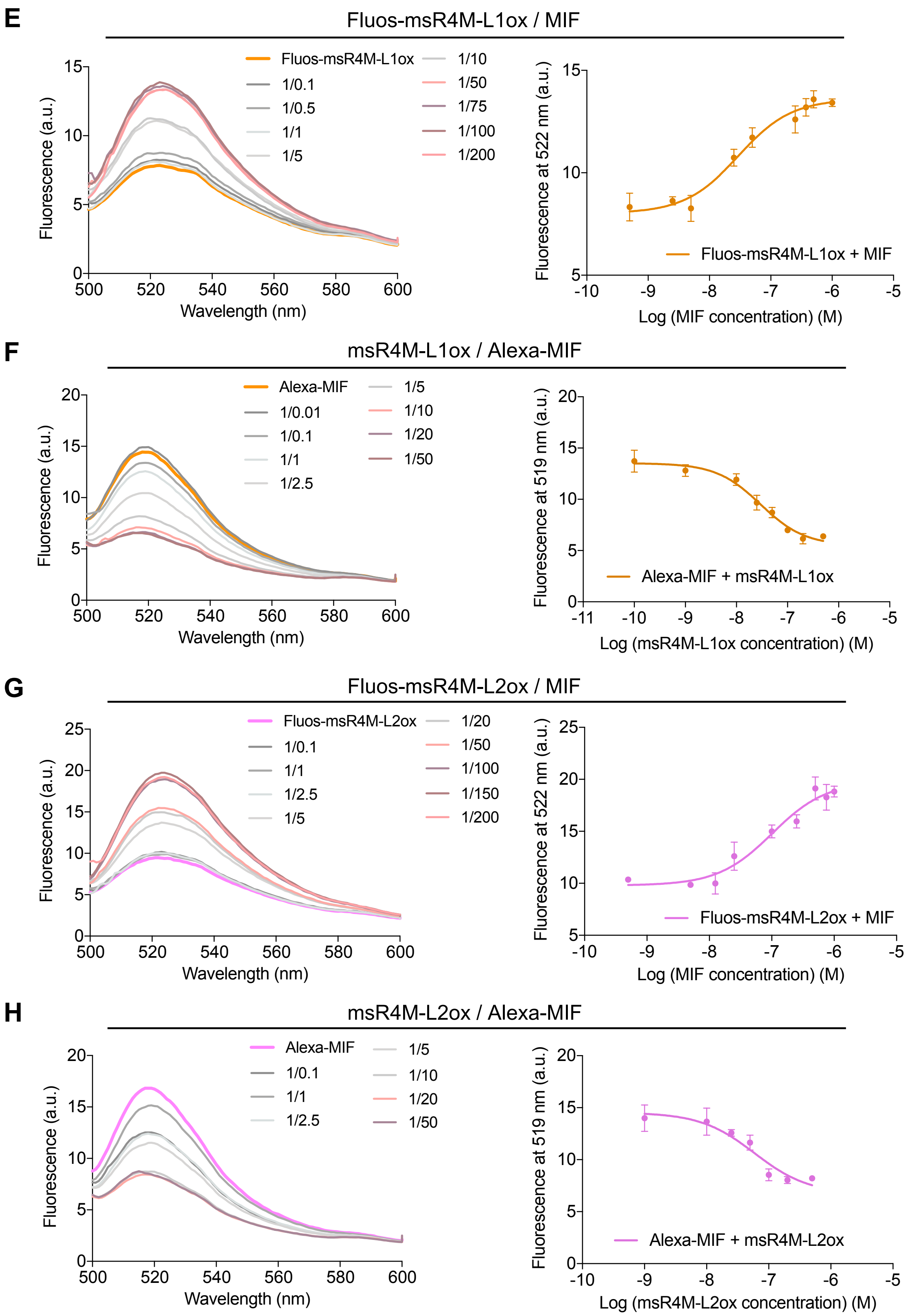
E
Fluos-msR4M-L1ox / MIF
Fluos-msR4M-L1ox
1/0.1
1/0.5
1/1
1/5
1/10
1/50
1/75
1/100
1/200
Fluorescence (a.u.)
Wavelength (nm)
Fluorescence at 522 nm (a.u.)
Fluos-msR4M-L1ox + MIF
Log (MIF concentration) (M)
F
msR4M-L1ox / Alexa-MIF
Alexa-MIF
1/0.01
1/0.1
1/1
1/2.5
1/5
1/10
1/20
1/50
Fluorescence (a.u.)
Wavelength (nm)
Fluorescence at 519 nm (a.u.)
Alexa-MIF + msR4M-L1ox
Log (msR4M-L1ox concentration) (M)
G
Fluos-msR4M-L2ox / MIF
Fluos-msR4M-L2ox
1/0.1
1/1
1/2.5
1/5
1/20
1/50
1/100
1/150
1/200
Fluorescence (a.u.)
Wavelength (nm)
Fluorescence at 522 nm (a.u.)
Fluos-msR4M-L2ox + MIF
Log (MIF concentration) (M)
H
msR4M-L2ox / Alexa-MIF
Alexa-MIF
1/0.1
1/1
1/2.5
1/5
1/10
1/20
1/50
Fluorescence (a.u.)
Wavelength (nm)
Fluorescence at 519 nm (a.u.)
Alexa-MIF + msR4M-L2ox
Log (msR4M-L2ox concentration) (M)

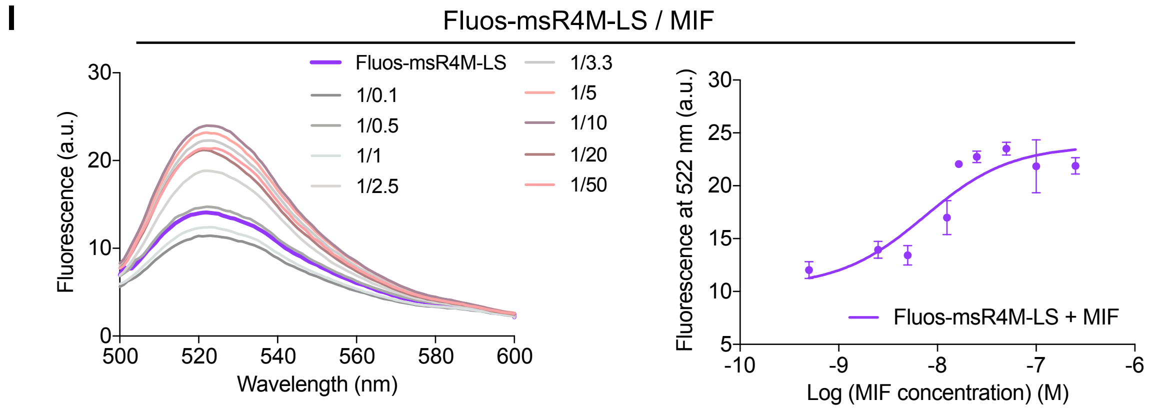
I
Fluos-msR4M-LS / MIF
Fluos-msR4M-LS
1/0.1
1/0.5
1/1
1/2.5
1/3.3
1/5
1/10
1/20
1/50
Fluorescence (a.u.)
Wavelength (nm)
Fluorescence at 522 nm (a.u.)
Fluos-msR4M-LS + MIF
Log (MIF concentration) (M)

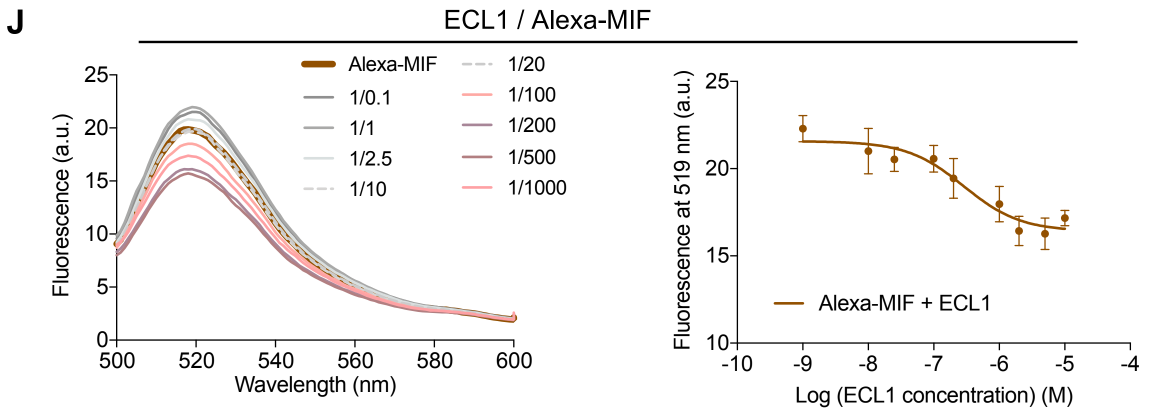
J
ECL1 / Alexa-MIF
Alexa-MIF
1/0.1
1/1
1/2.5
1/10
1/20
1/100
1/200
1/500
1/1000
Fluorescence (a.u.)
Wavelength (nm)
Fluorescence at 519 nm (a.u.)
Alexa-MIF + ECL1
Log (ECL1 concentration) (M)

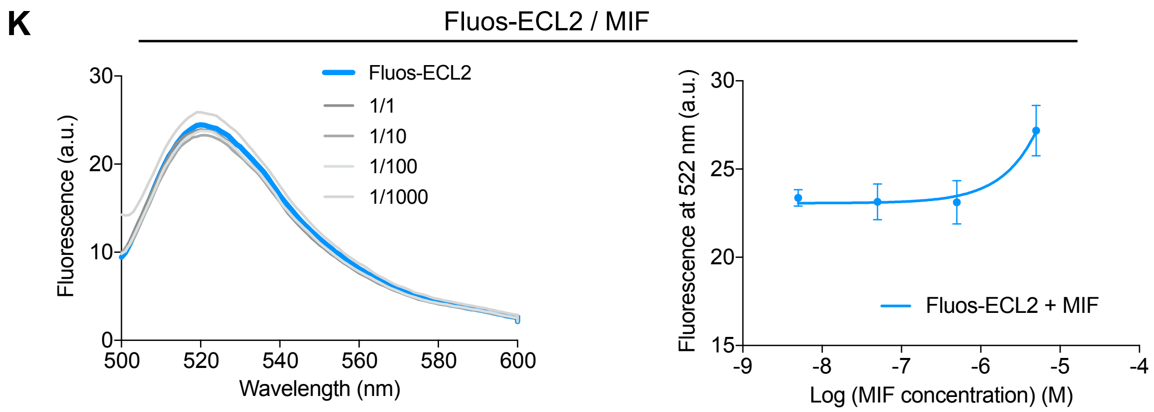
K
Fluos-ECL2 / MIF
Fluos-ECL2
1/1
1/10
1/100
1/1000
Fluorescence (a.u.)
Wavelength (nm)
Fluorescence at 522 nm (a.u.)
Fluos-ECL2 + MIF
Log (MIF concentration) (M)

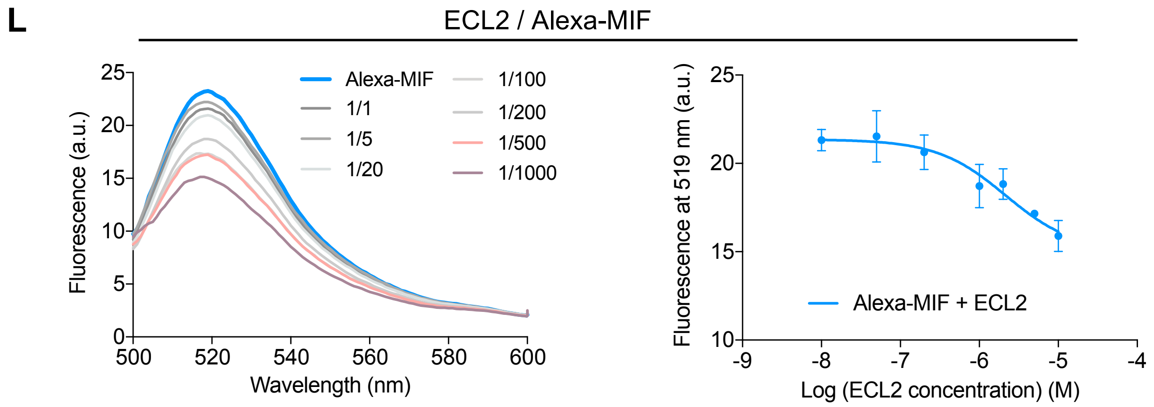
L
ECL2 / Alexa-MIF
Alexa-MIF
1/1
1/5
1/20
1/100
1/200
1/500
1/1000
Fluorescence (a.u.)
Wavelength (nm)
Fluorescence at 519 nm (a.u.)
Alexa-MIF + ECL2
Log (ECL2 concentration) (M)

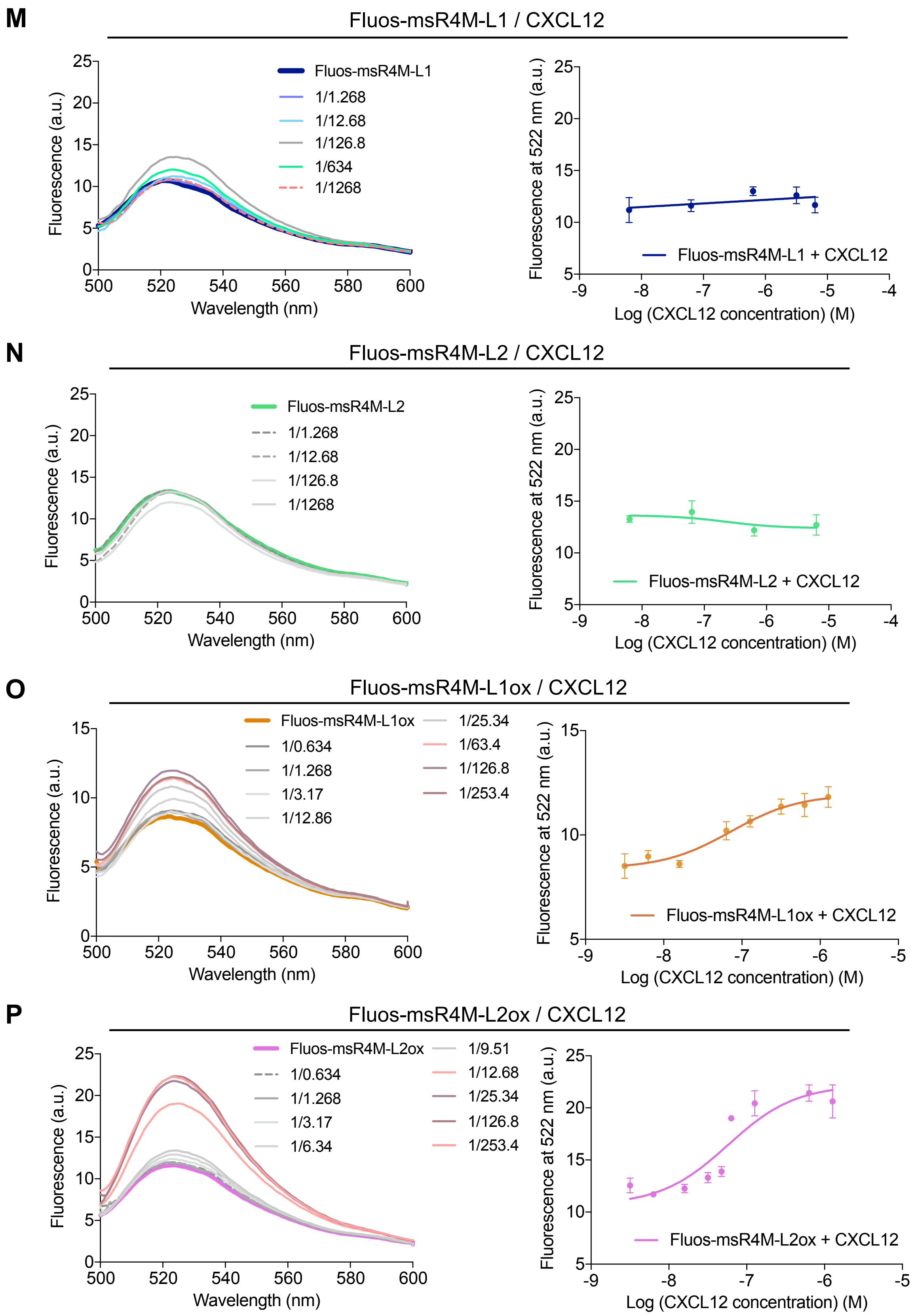
M
Fluos-msR4M-L1 / CXCL12
Fluorescence (a.u.)
Wavelength (nm)
Fluos-msR4M-L1
1/1.268
1/12.68
1/126.8
1/634
1/1268
Fluorescence at 522 nm (a.u.)
Log (CXCL12 concentration) (M)
Fluos-msR4M-L1 + CXCL12
N
Fluos-msR4M-L2 / CXCL12
Fluorescence (a.u.)
Wavelength (nm)
Fluos-msR4M-L2
1/1.268
1/12.68
1/126.8
1/1268
Fluorescence at 522 nm (a.u.)
Log (CXCL12 concentration) (M)
Fluos-msR4M-L2 + CXCL12
O
Fluos-msR4M-L1ox / CXCL12
Fluorescence (a.u.)
Wavelength (nm)
Fluos-msR4M-L1ox
1/0.634
1/1.268
1/3.17
1/12.86
1/25.34
1/63.4
1/126.8
1/253.4
Fluorescence at 522 nm (a.u.)
Log (CXCL12 concentration) (M)
Fluos-msR4M-L1ox + CXCL12
P
Fluos-msR4M-L2ox / CXCL12
Fluorescence (a.u.)
Wavelength (nm)
Fluos-msR4M-L2ox
1/0.634
1/1.268
1/3.17
1/6.34
1/9.51
1/12.68
1/25.34
1/126.8
1/253.4
Fluorescence at 522 nm (a.u.)
Log (CXCL12 concentration) (M)
Fluos-msR4M-L2ox + CXCL12

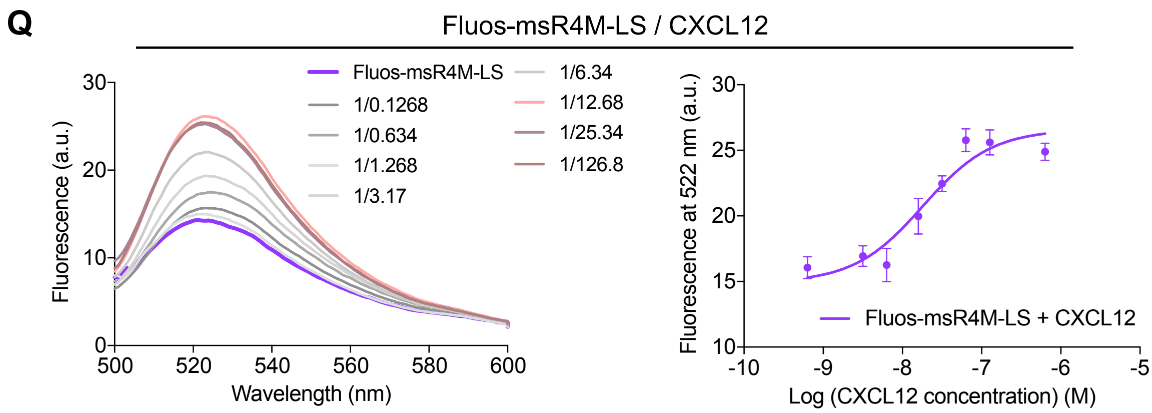


**Supplementary Figure 4** Binding affinities between CXCR4 ectodomain peptide mimics and MIF *versus* CXCL12 as determined by fluorescence spectroscopic titration. Fluorescently labeled (FLUOS) ectodomain peptides (linked peptides or single extracellular loop (ECL) peptides) were titrated with increasing concentrations of MIF or CXCL12; conversely for MIF, fluorescently labeled (Alexa-488) MIF was titrated with increasing concentrations of ecto-domain peptides. **Left panels of a-q** Fluorescence emission spectra. **Right panels of a-q** Binding curves derived from the fluorescence emission at 519 nm (Alexa-488 MIF) or 522 nm (Fluos-labeled peptides) as indicated. Binding affinities ($K_D$) were derived from these curves (see list of $K_D$ values in **Table 1** of the main manuscript). **a-l** Ectodomain peptide / MIF titrations as indicated; **m-q** ectodomain peptide / CXCL12 titrations; **a, c, e, g, i, k** Fluos-labeled ectodomain peptide / MIF titrations; **b, d, f, h, j, l** ectodomain peptide / Alexa-MIF titrations; **m-q** Fluos-labeled ectodomain peptide / CXCL12 titrations. Panels **a** and **m** are identical with **Fig. 1c** and **d** of the main manuscript, respectively, and are included in this Supplementary Figure for clarity reasons. Data shown are means ± SD from three independent titration experiments. Source data are provided as a Source Data file.

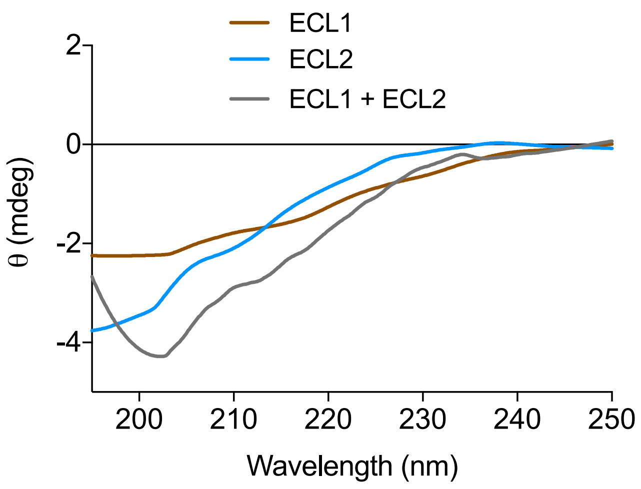


**Supplementary Figure 5** Circular dichroism (CD) spectroscopy reveals that non-linked mixtures of CXCR4 ectodomain loop sequences ECL1 and ECL2 exhibit more random coil contents than the linked mimics msR4M-L1 and msR4M-L2. CXCR4 ectodomain peptides ECL1[97-110] and ECL2[182-196] were mixed at a 1/1 ratio and subjected to far-UV CD spectroscopy. Corresponding individual ECL1 and 2 peptides were measured for comparison. CD spectra are represented as degrees of ellipticity ($\theta$, mdeg) as a function of the wavelength (given in nm) in the far-UV range. Source data are provided as a Source Data file.

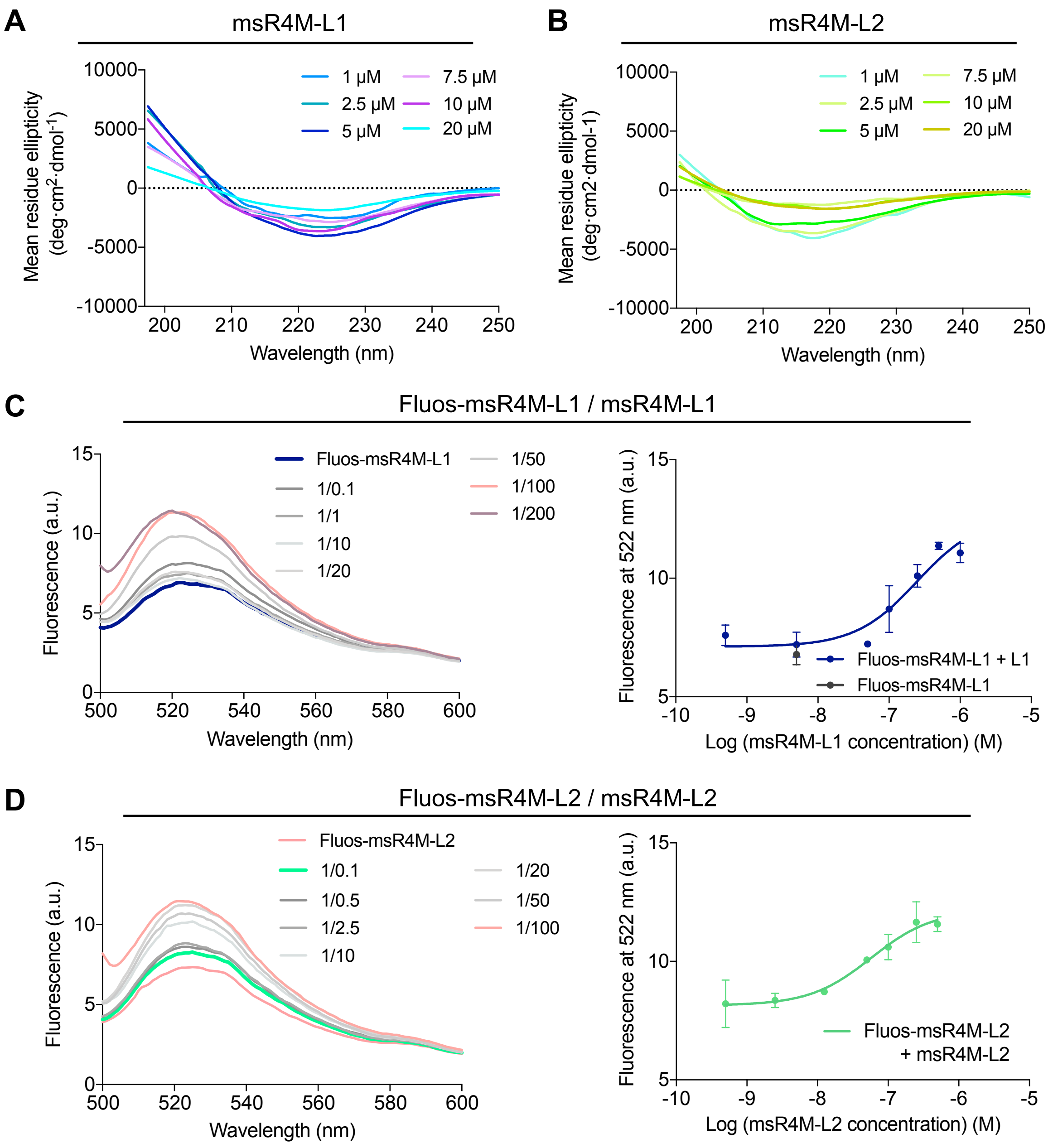


**Supplementary Figure 6** Self-assembly propensities of msR4M-L1 *versus* msR4M-L2 as determined by circular dichroism (CD) spectroscopy and fluorescence spectroscopic titration. **a, b** CD spectra of msR4M-L1 (**a**) and msR4M-L2 (**b**) at increasing concentrations (1, 2.5, 5, 7.5, 10 and 20 μM). The more pronounced concentration-dependent reduction in CD signal for msR4M-L2 (**b**) compared to msR4M-L1 (**a**) in the region between 210 and 225 nm indicates a higher aggregation propensity of msR4M-L2. Conformations in the CD spectra were measured as mean residue ellipticity (MRE) as a function of the wavelength (given in nm) in the far-UV range. **c**, **d** Determination of self-assembly propensity by fluorescence titration spectroscopy. Binding ('self-assembly') was analyzed by titrating fluorescently labeled (Fluos) msR4M-L1 and msR4M-L2 with increasing concentrations of unlabeled msR4M-L1 (**c**) and msR4M-L2 (**d**), respectively. **Left panels of c**,**d** Fluorescence emission spectra. **Right panels of c**,**d** Binding curves derived from the fluorescence emission at 522 nm. Data are means ± SD of three independent titration experiments. Source data are provided as a Source Data file.

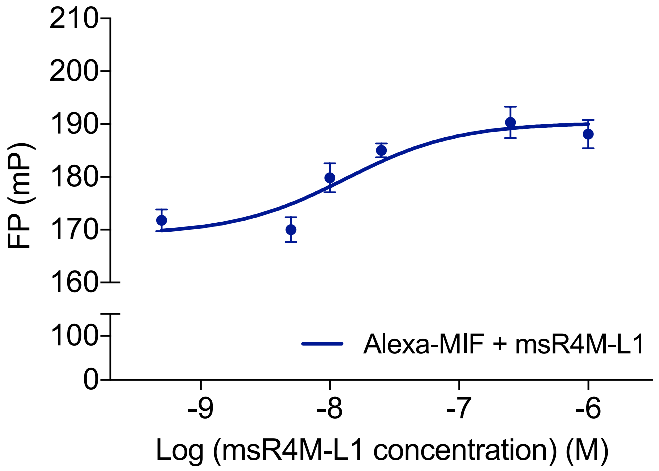


**Supplementary Figure 7** Binding between MIF and msR4M-L1 as determined by fluorescence polarization (FP). Alexa-MIF was titrated against increasing concentrations of msR4M-L1. Changes in FP were recorded at 519 nm and are indicated as fluorescence polarization (FP (mP)). Data are means ± SD of three independent titration experiments. Source data are provided as a Source Data file.

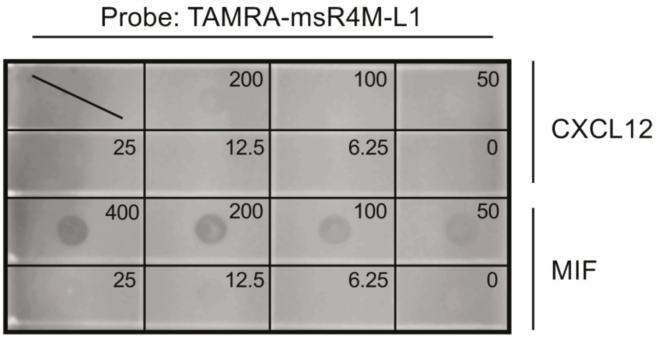


**Supplementary Figure 8** Binding analysis between TAMRA-msR4M-L1 and MIF *versus* CXCL12 as determined by dot blot titration. A representative blot of a total of three independent experiments is shown. For quantification of the three blots, see Fig. 1j.

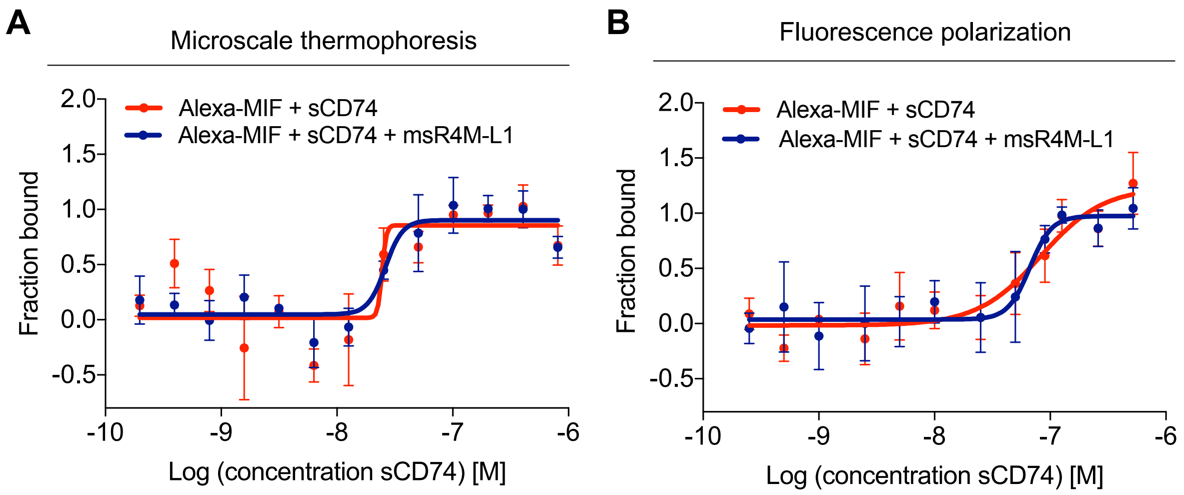


**Supplementary Figure 9** msR4M-L1 does not affect the binding between MIF and soluble CD74 as analyzed by microscale thermophoresis (MST) and fluorescence polarization (FP). **a** Microscale thermophoresis. Before titrating Alexa-647-MIF (Alexa-MIF, 20 nM) against HA-tagged soluble CD74(73-232) (sCD74), Alexa-MIF was either left untreated (red curve) or was pre-incubated with a 10-fold molar excess of msR4M-L1 (blue curve). Changes were recorded as ΔFnorm(‰) and binding between MIF and sCD74 in the absence (red) *versus* presence (blue) of msR4M-L1 is plotted as fraction bound. Data are means ± SD of three independent titration experiments. **b** Fluorescence polarization. Same as **a**, except that Alexa-488-MIF (10 nM) was used instead of Alexa-647-MIF (both abbreviated as Alexa-MIF in their respective graphs) and that a 20-fold molar excess of msR4M-L1 was used. Changes in FP (mP) were recorded at 519 nm and binding curves in the absence *versus* presence of msR4M-L1 are indicated as fraction bound. Data are means ± SD of three independent titration experiments. Source data are provided as a Source Data file.

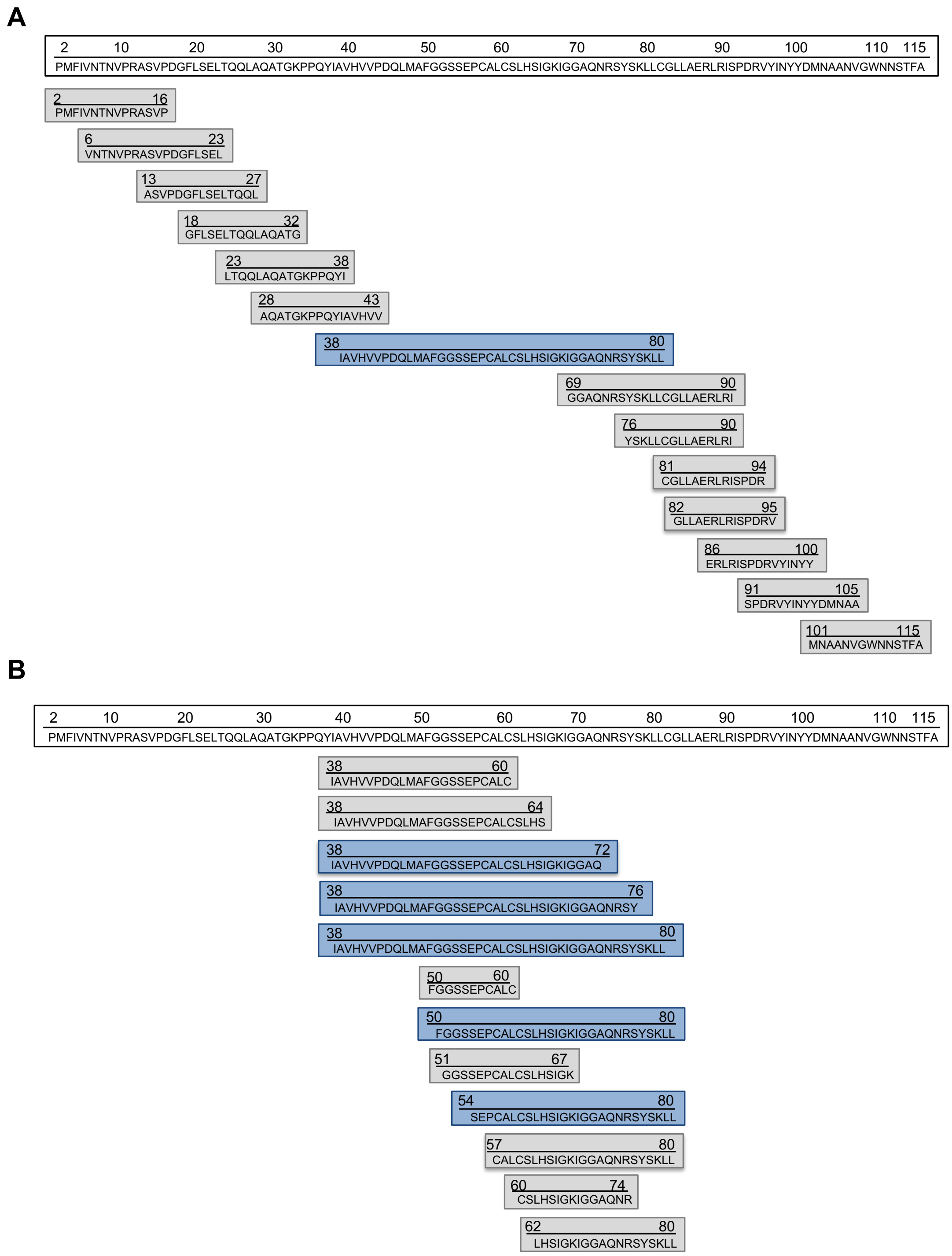


**Supplementary Figure 10** Overview of the MIF sequences involved in binding to the CXCR4 ectodomain peptide msR4M-L1. MIF sequences with high binding affinity are colored in blue, non-binders/low affinity-binders are depicted in grey. The scheme visualizes the binding affinities ($K_D$) between msR4M-L1 and partial MIF peptides as determined by fluorescence titration spectroscopy and listed in detail in **Supplementary Table 2**. **a** Coverage of the full-length MIF sequence highlighting the core binding region 38-80 (blue) and the tested, non-binding, peptides located N- and C-terminal of this region (grey). **b** Focus on region 38-80. Binding (blue) versus non-binding/low-affinity binding (grey) partial MIF sequences within this region are aligned. For the purpose of this overview scheme, binding is defined relative to the affinity between msR4M-L1 and full-length MIF: binding/high-affinity binding as "within a range of 5-10 x $K_D$ of msR4M-L1" and non-binding/low-affinity binding as "more than 100 x higher $K_D$ than the one of msR4M".

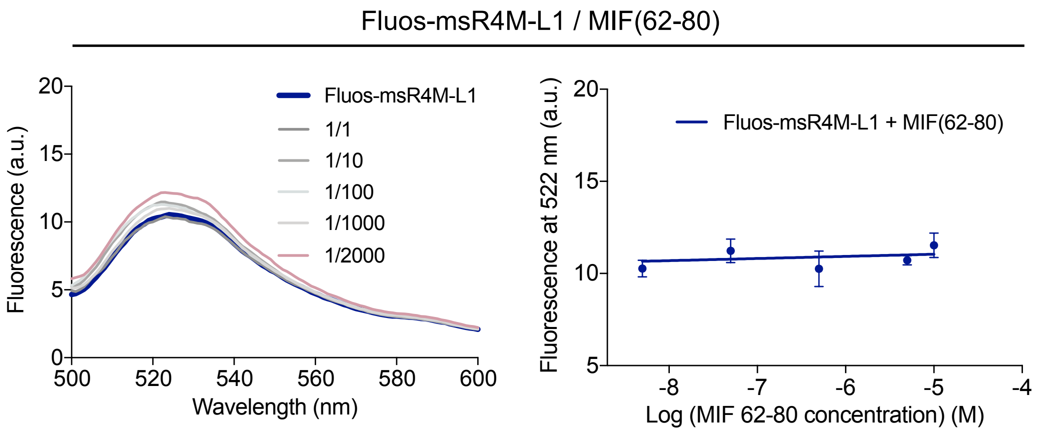


**Supplementary Figure 11** Engineered CXCR4 mimic msR4M-L1 does not bind MIF fragment MIF(62-80) that is located at the C-terminal end of the MIF core region. Fluorescence spectroscopic titration of fluorescently labeled (Fluos) msR4M-L1 (5 nM) with increasing concentrations of MIF(62-80). Left, fluorescence emission spectra; right, binding curve ($K_D$ >10 µM). Data shown are means ± SD from three independent titration experiments. Source data are provided as a Source Data file.

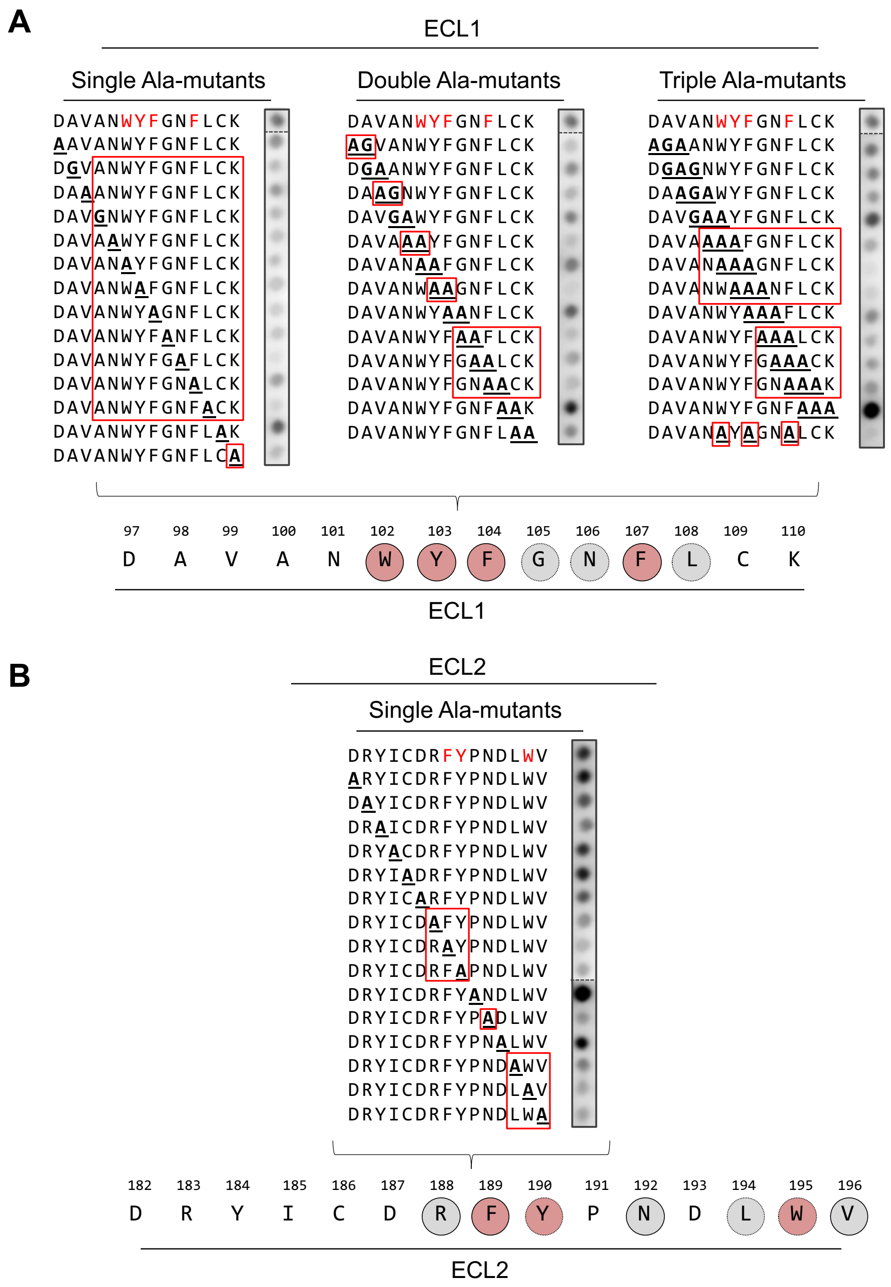


**Supplementary Figure 12** Peptide array analysis in combination with alanine-scanning identifies aromatic residues within msR4M-L1 critical for MIF binding. 14-15-meric peptides of relevant ECL1 (**a**) and ECL2 (**b**) sequences were synthesized and immobilized on glass slides. Residues were substituted by alanine as indicated (bold, underlined). Alanine residues in native sequences were replaced by glycine (A→G). Dashed lines on array indicate that spots were from different rows on the 384-spot array and aligned here for better overview. Arrays were probed with biotinylated MIF; spot intensity indicates binding strength between a respective peptide and MIF. In ECL2, a single alanine-scan pinpointed to only seven critical residues including three aromatic residues in positions 189, 190, and 195, for which strongest reduction in MIF binding was seen upon substitution by Ala. For ECL1, the single Ala-mutants were not as conclusive, but analysis of double and triple Ala-mutants narrowed down binding-determining residues to a few positions including four critical aromatic residues in position 102, 103, 104, and 107. The sequence at the bottom panel of **a** and **b** summarizes the result of the scans: red circle, critical aromatic residue; grey circle, other relevant residue; no circle, no or low contribution to MIF binding. Ala-substitutions that led to significant reduction in binding are boxed in red. Critical aromatic residues substituted by Ala in msR4M-L1(7xAla) are colored or circled in red (first sequence row, summary line, respectively). Source data are provided as a Source Data file.

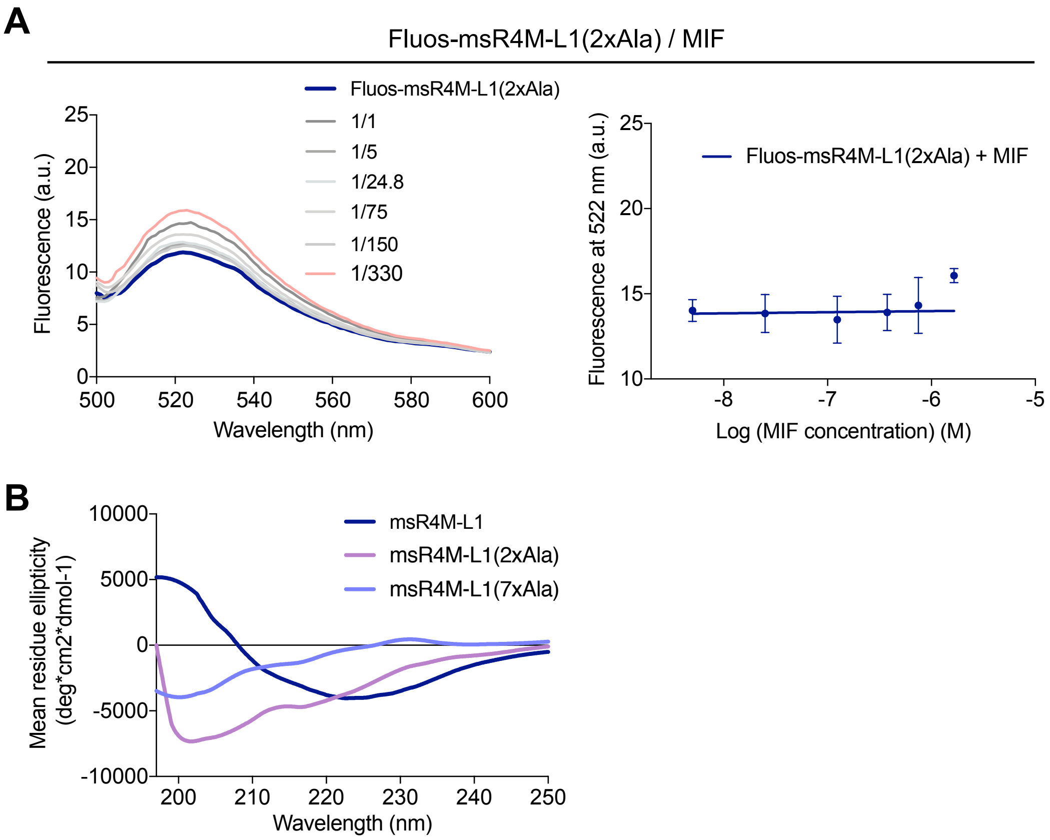


**Supplementary Figure 13** Fluorescence spectroscopic binding data and circular dichroism spectroscopy (CD) of alanine mutants of msR4M-L1. **a** msR4M-L1(2xAla), in which Phe-104 and Phe-107 residues are substituted by alanine, does not bind to MIF as indicated by fluorescence spectroscopy titration. Fluorescently labeled (Fluos) msR4M-L1(2xAla) was titrated with increasing concentrations of MIF. Left, Fluorescence emission spectra; right, binding curve derived from the fluorescence emission at 522 nm; binding affinity (app. $K_D$ >1.5 µM). Data shown are means ± SD from three independent titration experiments. **b** Alanine substitution of specific aromatic residues within msR4M-L1 leads to loss of structure. CD spectra of msR4M-L1(7xAla) and msR4M-L1(2xAla) and “wildtype” msR4M-L1 for comparison (5 µM each). Substitution of all aromatic residues in msR4M-L1(7xAla) leads to a nearly complete loss of structure, while the 2xAla analog retains some structural elements. CD spectra show mean residue ellipticities (MRE) as a function of the wavelength (nm) in the far-UV range. Source data are provided as a Source Data file.

**A**

```
                                                                            54-80
                                                              38-80
        2
Human   PMFIVNTNVPRASVPDGFLSELTQQLAQATGKPPQYIAVHVVPDQLMAFGGSSEPCALCSLHSIGKIGGAQNRSYSKLLCG
Mouse   PMFIVNTNVPRASVPEGFLSELTQQLAQATGKPAQYIAVHVVPDQLMTFSGTNDPCALCSLHSIGKIGGAQNRNYSKLLCG
        ***************:***************** *************:*.*:.:*******************.*******

                                            115
Human   LLAERLRISPDRVYINYYDMNAANVGWNNSTFA
Mouse   LLSDRLHISPDRVYINYYDMNAANVGWNGSTFA
        **::**:*********************.****
```

**B**

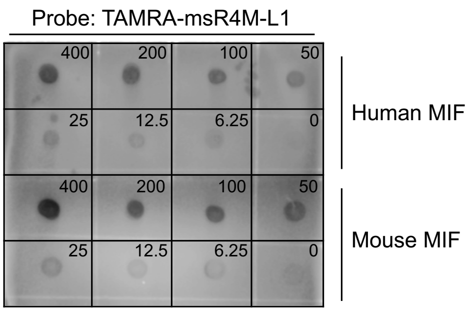


**Supplementary Figure 14** High degree of sequence identity between human and mouse MIF and equal binding affinity of msR4M-L1 to human and mouse MIF. **a** Sequence comparison between human and mouse MIF across the full protein sequence. The high degree of sequence identity between human and mouse MIF is also represented in the core msR4M-L1 binding region at sequence 38-80 or 54-80. Sequence alignment between the protein sequences of human and mouse MIF using the multiple sequence alignment tool ClustalW2 (EMBL-EBI, https://www.ebi.ac.uk/Tools/msa/clustalw2/). Conserved residues are marked with an asterisk (*), conservative replacements by a colon (:) and blue color, semi-conservative replacements by a half-colon (.) and green color, and non-conservative replacements by empty denotation ( ) and red color. **b** Dot blot demonstrates that msR4M-L1 binds equally well to human and mouse MIF. Different protein amounts of human and mouse MIF were spotted as indicated and blots developed with TAMRA-labeled msR4M-L1. The blot shown is representative of two independently performed experiments.

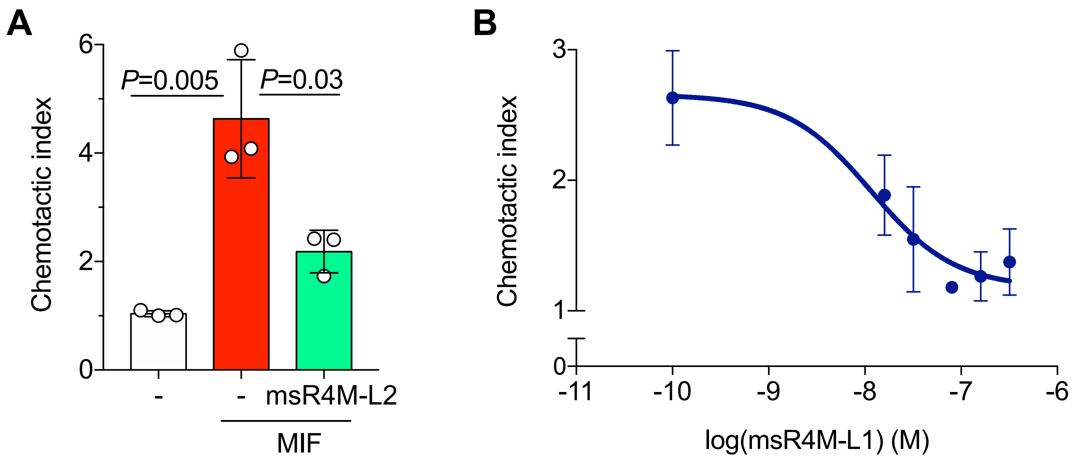


**Supplementary Figure 15** Inhibition of MIF-triggered primary mouse B-lymphocyte chemotaxis by msR4M-L2 and msR4M-L1. MIF was added to the lower chamber of a Transwell device as chemoattractant at a concentration of 16 nM. **a** msR4M-L2 inhibits MIF-triggered B-lymphocyte chemotaxis. msR4M-L2 was added at a 5-fold molar excess over MIF. **b** Concentration-dependent inhibition of MIF-mediated B-lymphocyte chemotaxis by msR4M-L1. The chemotactic index of MIF-elicited chemotaxis is plotted against the different concentrations of msR4M-L1. IC50 plot with the chemotactic index plotted against the log(concentration) of msR4M-L1. The deduced estimated IC50 value is 10 nM. The data shown are means ± SD of three experiments. Statistical analysis (**a**) was performed by unpaired two-tailed T-test; *P* values are indicated. Source data are provided as a Source Data file.

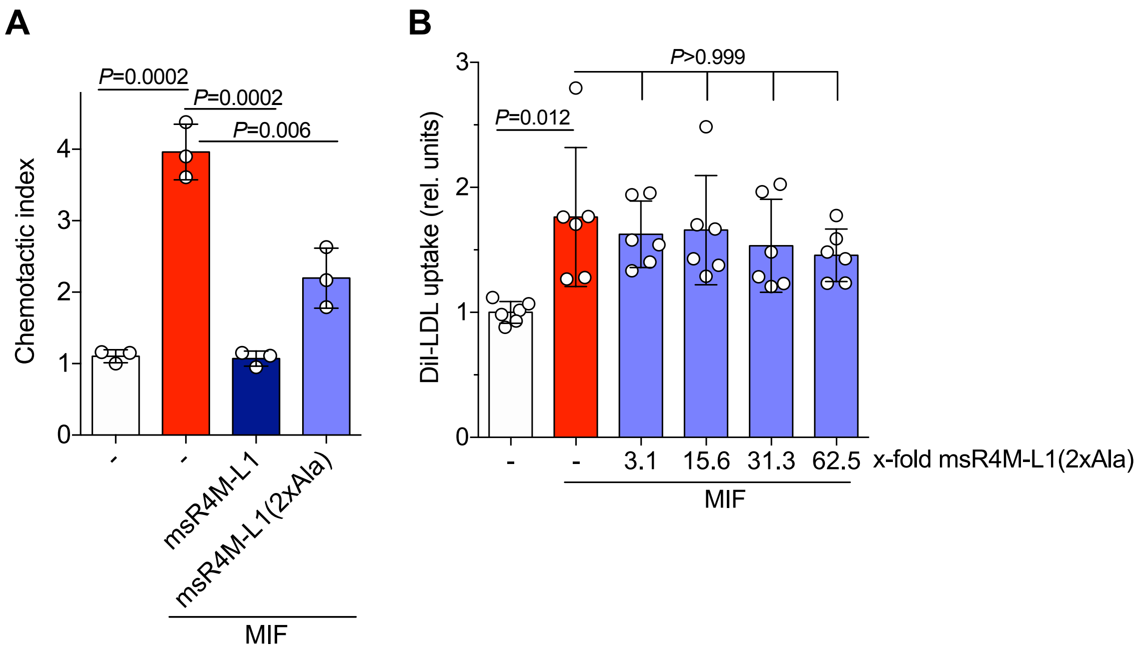


**Supplementary Figure 16** Inhibition by msR4M-L1 of MIF-triggered atherogenic activity *in vitro* is (partially) abrogated by substituting two residues within the msR4M-L1 binding core with alanine. **a** Compared to msR4M-L1, inhibition of MIF-triggered primary mouse B-lymphocyte chemotaxis by msR4M-L1(2xAla) is partially reversed. MIF was added to the lower chamber of a Transwell device as chemoattractant at a concentration of 16 nM. msR4M-L1 and msR4M-L1(2xAla) were added at a 5-fold molar excess over MIF. The chemotactic index of MIF-elicited chemotaxis is plotted against the different concentrations of CXCR4 ectodomain mimic or its mutant. The data shown are means ± SD of three chemotaxis assays with B cells from one mouse preparation. Statistical analysis was performed by unpaired two-tailed T-test; *P* values are indicated. **b** msR4M-L1(2xAla) fails to inhibit MIF-elicited uptake of DiI-LDL by human macrophages. MIF-dependent DiI-LDL uptake in primary human monocyte-derived macrophages as read out by microscopic quantification of DiI-LDL-positive cells. MIF was applied at a concentration of 80 nM. Concentration of msR4M-L1(2xAla) is indicated as molar excess over MIF. Quantification is from three-times-two independent experiments; 9 fields-of-view each). The data shown are means ± SD. Statistical analysis was performed with unpaired two-tailed T-test or Kruskal-Wallis test with Dunn's multiple comparisons test; *P* values are indicated. For the msR4M-L1(7xAla) mutant, see **Fig. 3** and **4** of the main manuscript. Source data are provided as a Source Data file.

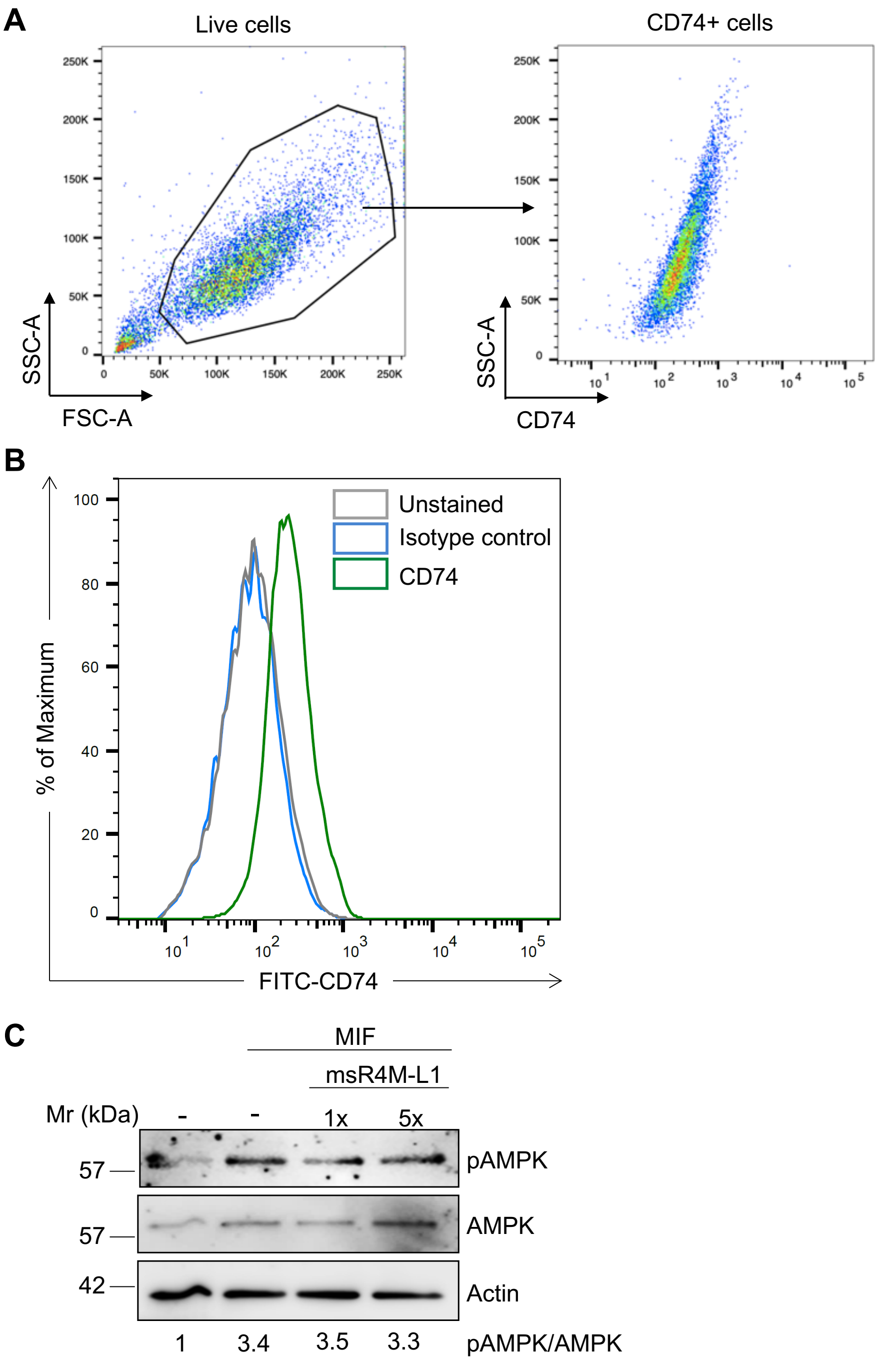


**Supplementary Figure 17** msR4M-L1 does not interfere with MIF-triggered AMPK signaling in the CD74-expressing human cardiomyocyte cell line HCM. **a-b** Human cardiac myocytes (HCMs) express CD74 on their surface. **a** HCM gating scheme indicating live (side (SSC) *versus* forward (FSC) scatter) and CD74-expressing (CD74+) cells (SSC *versus* CD74+). **b** Flow cytometry verified marked surface expression of CD74 on HMCs. A FITC-conjugated anti-CD74 antibody (green) was used for detection. FITC-IgG2 (isotype control, blue) and cell incubations without antibody were measured as negative controls. **c** msR4M-L1 does not interfere with MIF-triggered AMPK signaling. MIF was applied at a concentration of 16 nM; msR4M-L1 was added at 1- and 5-fold molar excess over MIF. A representative Western blot (from a total of five independent experiments) from HCM lysates developed against pAMPK and total AMPK is shown. The blot was quantified by densitometry of pAMPK normalized against AMPK; the signal intensity expressed as pAMPK/AMPK is indicated. Actin is shown as additional loading control. Molecular weight markers (CozyHi™ prestained ladder, highQu, Kraichtal, Germany) were run in the same gel and are indicated. Source data are provided as a Source Data file.

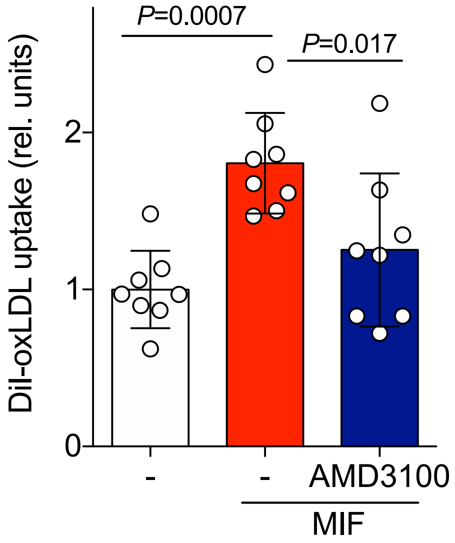


**Supplementary Figure 18** The MIF-mediated increase in oxLDL uptake by macrophages is dependent on CXCR4 – evidence from inhibition with AMD3100. DiI-oxLDL uptake in was measured in primary human monocyte-derived macrophages. MIF was applied at a concentration of 80 nM and AMD3100 was used at a concentration of 10 µM. The data shown are means ± SD from n = 4 independent biological experiments with two independent incubations each. Statistical analysis was performed with one-way ANOVA with Tukey's multiple comparison; *P* values are indicated. Source data are provided as a Source Data file.

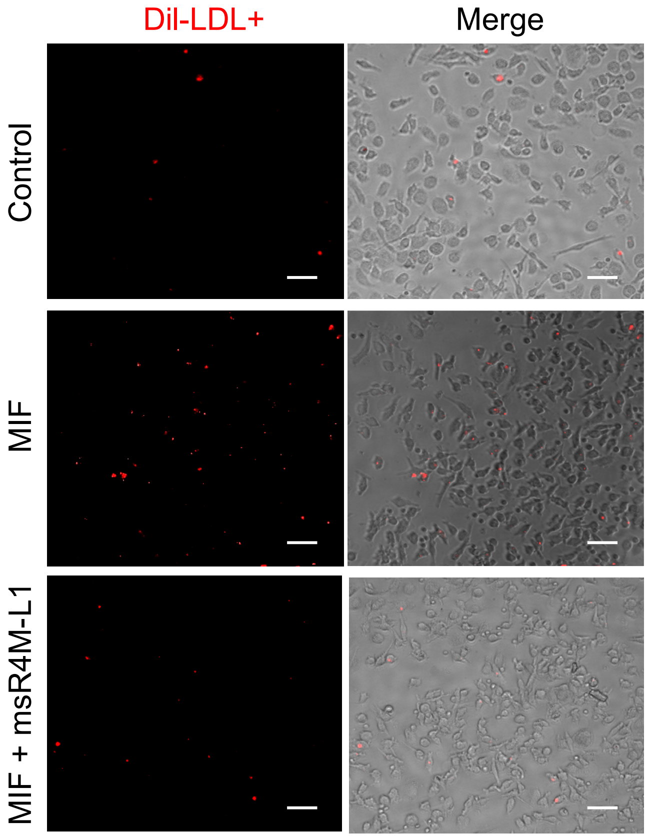


**Supplementary Figure 19** CXCR4 ectodomain mimic msR4M-L1 inhibits MIF-elicited DiI-LDL uptake in primary human monocyte-derived macrophages. Representative images of DiI-LDL-positive cells (DiI-LDL+) are shown from macrophages treated with MIF or MIF + msR4M-L1 and compared to control buffer. The images are representative of four-times-two independent experiments with 9 fields-of-view analyzed each. MIF was applied at a concentration of 80 nM and msR4M-L1 at 20x molar excess over MIF. Left panel, DiI fluorescence; right panel, merge with phase contrast to visualize cells bodies. Scale bar: 50 µm.

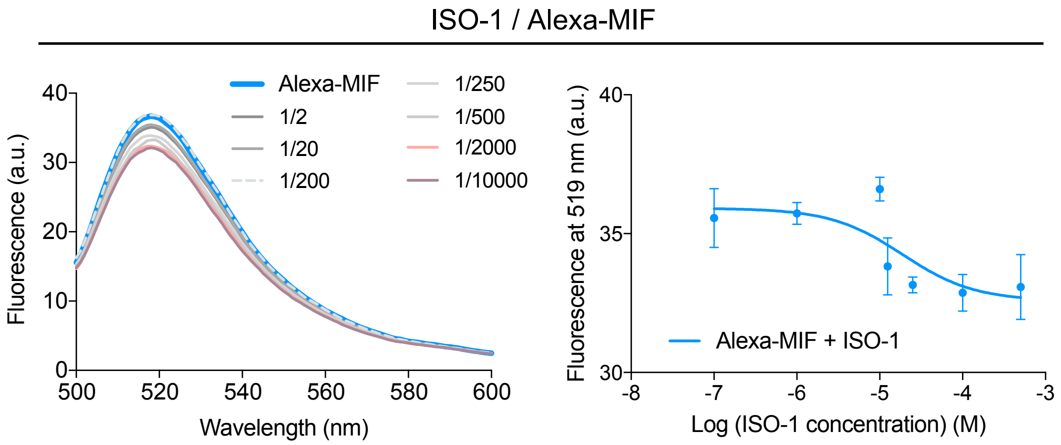


**Supplementary Figure 20** Binding affinity between MIF and the small molecule MIF inhibitor ISO-1 as determined by fluorescence titration spectroscopy. Emission spectra of Alexa-MIF alone (blue) and with increasing concentrations of ISO-1 at indicated ratios are shown (**left panel**; representative titration); binding curve derived from the fluorescence emission at 519 nm (**right panel**). The derived binding affinity (app. $K_D$) is 14.4±4.4 μM. The data shown are means ± SD of n = 3 experiments. Source data are provided as a Source Data file.

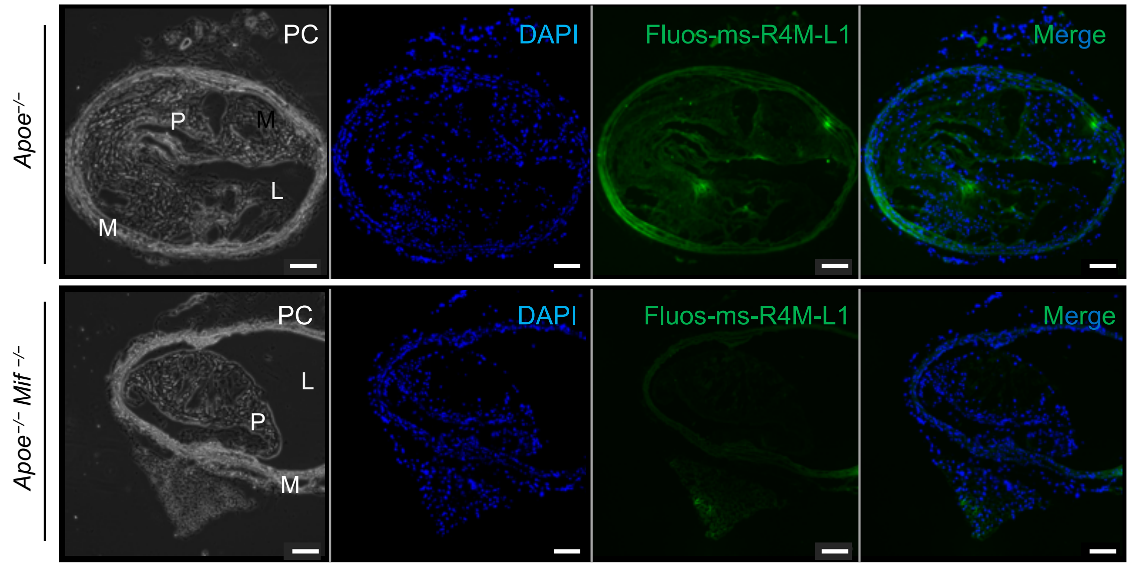


**Supplementary Figure 21** Fluos-msR4M-L1 stains atherosclerotic plaque tissue from atherogenic mice in a MIF-selective manner. Cryosections of the atherosclerotic predilection site of brachiocephalic artery (BCA) from atherogenic *Mif*-expressing *Apoe*$^{-/-}$ (top) *versus Mif*-deficient *Apoe*$^{-/-}$*Mif*$^{-/-}$ (bottom) on Western-type high-fat diet (HFD) were prepared for immuno-fluorescent staining and incubated with 500 nM Fluos-msR4M-L1. DAPI was used for counter-staining. BCA sections from *Apoe*$^{-/-}$*Mif*$^{-/-}$ only show background staining, indicating MIF selectivity of the Fluos-msR4M-L1 positivity. The experiment shown is representative of two independent experiments. PC, phase contrast image of tissue section; P, plaque area; L, lumen; M, media. Scale bar: 200 µm.

A

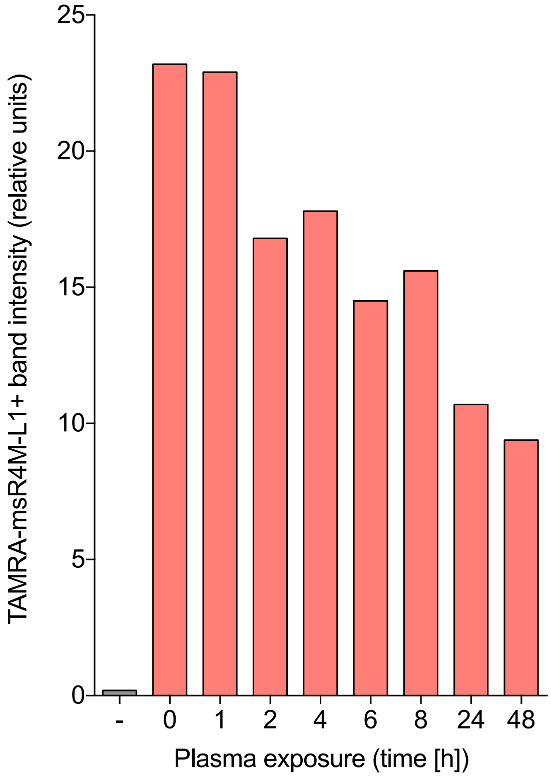


B

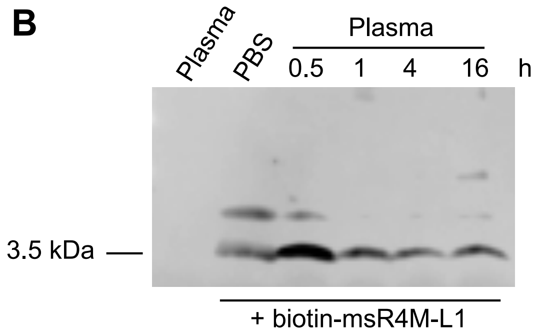


**Supplementary Figure 22** msR4M-L1 exhibits reasonable proteolytic stability in mouse and human plasma. **a** msR4M-L1 exhibits reasonable stability in mouse plasma. Densitometric quantification of TAMRA-msR4M-L1 bands following SDS-PAGE analysis of mouse plasma samples, in which 125 ng TAMRA-msR4M-L1 (corresponding to a concentration of 482 nM) had been incubated for the indicated time intervals at 37°C (orange/red bars). Gels were imaged for red TAMRA fluorescence with an Odyssey® Fc imager. The SDS-PAGE gel from the densitometry was performed is depicted in **Fig. 6a** of the main manuscript. The densitometry data represent a single reading from one SDS-PAGE gel and are representative of two independent experiments. Grey bar, a plasma sample without peptide was electrophoresed as control. **b** msR4M-L1 exhibits reasonable stability in human plasma. SDS-PAGE/Western blot analysis of human plasma samples, in which 583 ng of biotin-msR4M-L1 (corresponding to a concentration of 13.7 µM) had been incubated for the indicated time intervals at 37°C. The Western blot was developed with streptavidin-POD. PBS, one-hour incubation of biotin-msR4M-L1 in PBS as control; plasma alone, control incubation without peptide. Source data are provided as a Source Data file.

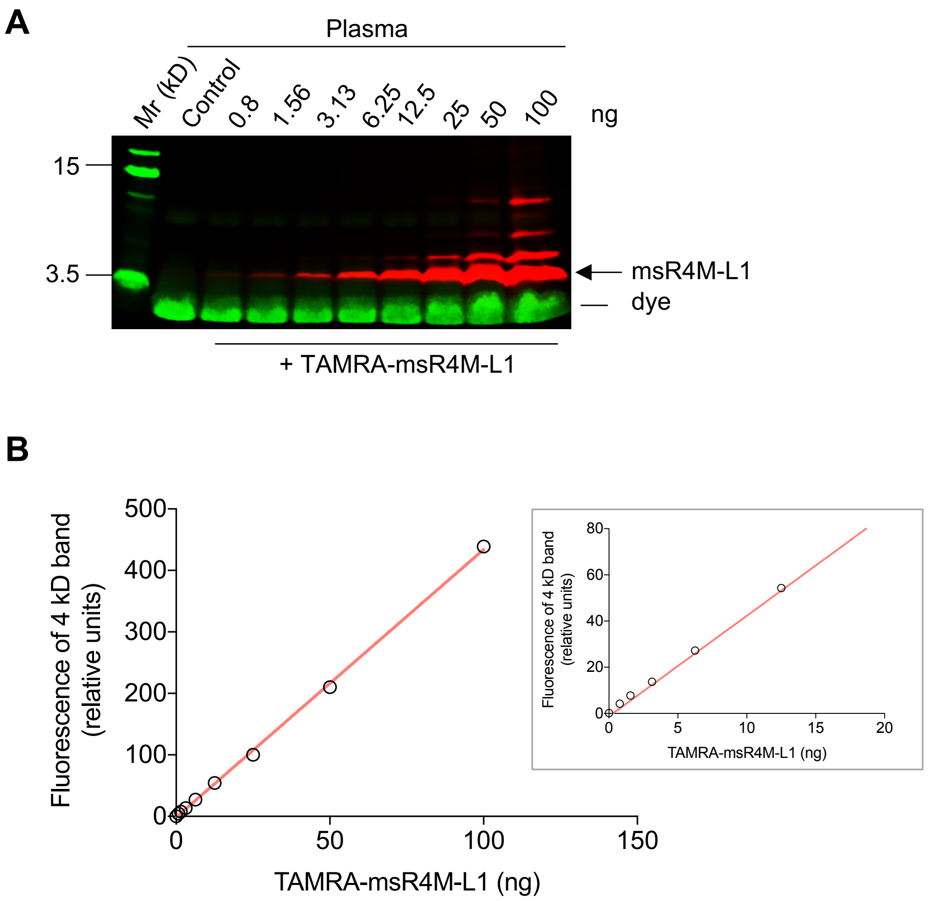


**Supplementary Figure 23** Dose calibration of TAMRA-msR4M-L1 in mouse plasma. **a** SDS-PAGE analysis of TAMRA-msR4M-L1 in mouse plasma. The indicated amounts of TAMRA-msR4M-L1 were dissolved in mouse plasma, incubated for 60 min and 37°C, mixed with SDS-PAGE sample buffer and electrophoresed in a 10-20% Tricine gel. Net amounts of TAMRA-msR4M-L1 loaded per lane are indicated. The TAMRA-msR4M-L1 band was visualized by red fluorescence imaging using an Odyssey® Fc imager. Control, plasma without TAMRA-mR4M-L1; dye, the running front dye is colored in green. The gel shown is representative of two independent experiments. **b** Quantification of the gel in **a**. The red fluorescence of the 4 kD msR4M-L1 band was quantified as relative fluorescence units at each loaded amount of TAMRA-msR4M-L1 by Odyssey® Fc imager and linear regression analysis applied. Inset, extension of the dose range between 0 and 12.5 ng. Source data are provided as a Source Data file.

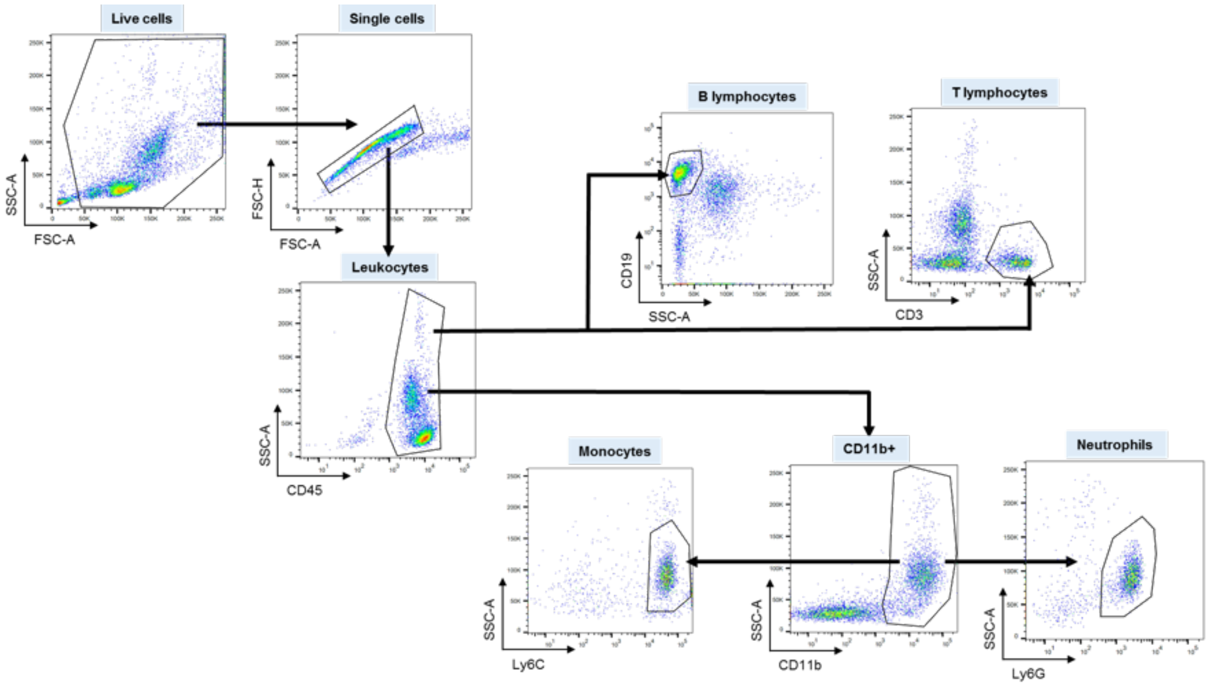


**Supplementary Figure 24** Gating strategy for blood cell analysis by FACS. Blood was from *Apoe*$^{-/-}$ mice on cholesterol-rich high-fat diet (HFD) for 4.5 weeks. For details of the blood cell analysis, see Supplementary Table 3. Leukocyte subsets were gated as indicated. SSC-A, side scatter area; FSC-A, forward scatter area; FSC-H, forward scatter height.

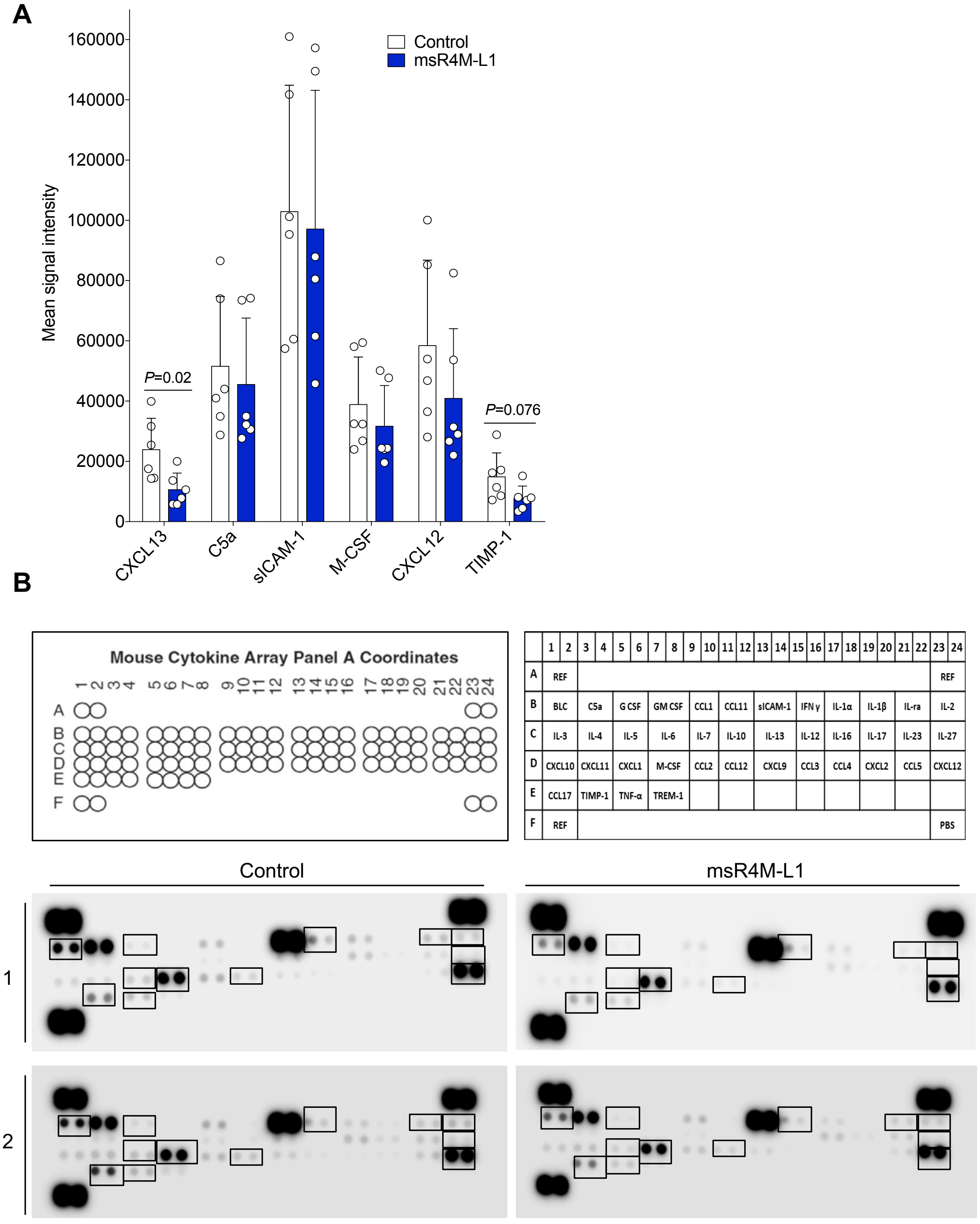


**Supplementary Figure 25** Therapeutic administration of msR4M-L1 in atherogenic *Apoe*$^{-/-}$ mice leads to a reduction in circulating inflammatory cytokine levels. Mouse cytokine array panel A (R&D Systems) featuring 40, mostly inflammatory/atherogenic, cytokines/chemokines was performed on plasma samples from *Apoe*$^{-/-}$ mice on HFD that were therapeutically treated with msR4M-L1 or vehicle control according to **Fig. 6b**. The quantitative analysis of 34 of these cytokines/chemokines is shown in **Fig. 6i**. **a** Quantitative analysis of the levels of the remaining 6 cytokines/chemokines of the panel, i.e. those that have much higher circulating levels and give much higher signals on the developed array membrane. **b** Array set-up (***top***) and images of the developed arrays (***bottom***). The experiment shown represents the analysis of two out of six mice from each cohort (labelled 1, 2); each array analysis was performed in duplicate. Statistics are reported as means ± SD from 6 independent experiments, corresponding to 6

mice per group, performed in duplicate each. Statistical analysis (**a**) was performed by multiple two-tailed T-test or two-tailed Mann-Whitney test as appropriate. The *P* value for CXCL13 ($P = 0.02$) is indicated, as well as that for TIMP-1, which shows a trend towards reduction ($P = 0.076$). The differences for the 4 other cytokines were not significant. Cytokines/chemokine names are used according to the panel nomenclature. BLC, CXCL13; C5a, complement factor 5a; G-CSF, granulocyte-colony-stimulating factor; GM-CSF, granulocyte-macrophage-CSF; sICAM-1, soluble ICAM-1; IFN-y, interferon-gamma; IL-1ra, IL-1 receptor antagonist; CCL/CXCL, CC-type and CXC-type chemokine; M-CSF, monocyte-CSF; IL, interleukin; TIMP-1, tissue inhibitor of metalloproteinases-1; TNF-$\alpha$, tumor necrosis factor-a; TREM-1, soluble form of triggering receptor expressed on myeloid cells-1. Source data are provided as a Source Data file.

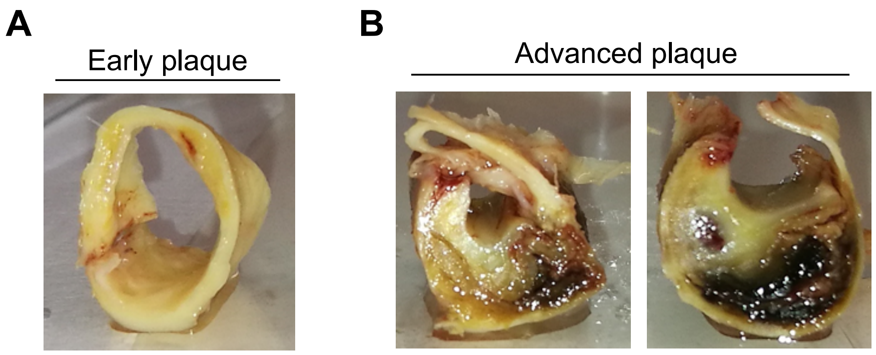


**Supplementary Figure 26** Human carotid atherosclerotic plaque tissue from patients who underwent carotid endarterectomy (CEA); representative examples of carotid specimens with early (one example, left) and advanced (two examples, right) plaques as used at Munich Vascular Biobank. Comparable specimens were used for the fresh sample qPCR and RNAseq analyses in Fig. 6.

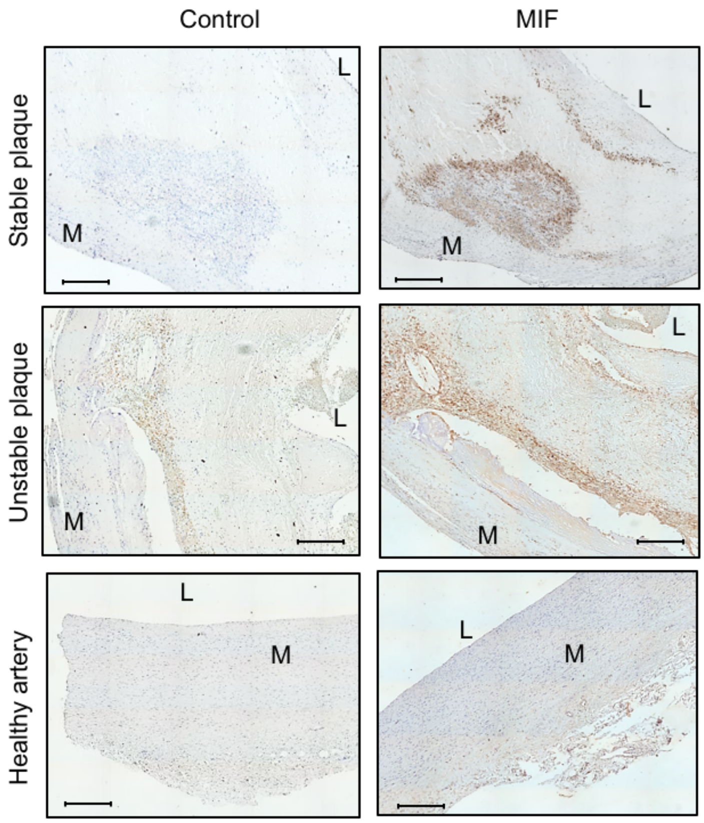


**Supplementary Figure 27** MIF protein expression in human carotid atherosclerotic plaque tissue from patients who underwent carotid endarterectomy (CEA). Primary antibody control. MIF was detected by DAB-based immunohistochemistry (IHC) using a polyclonal anti-MIF antibody (MIF) and counterstaining with Mayer hematoxylin. Representative images from a n=3 stable plaques, n=3 unstable plaques, and n=3 healthy control vasculature specimens are shown. Control stainings were performed in the absence of primary antibody (control). Size bars are 500 µm. Plaque/vessel regions are indicated for orientation (L, lumen; M, media).

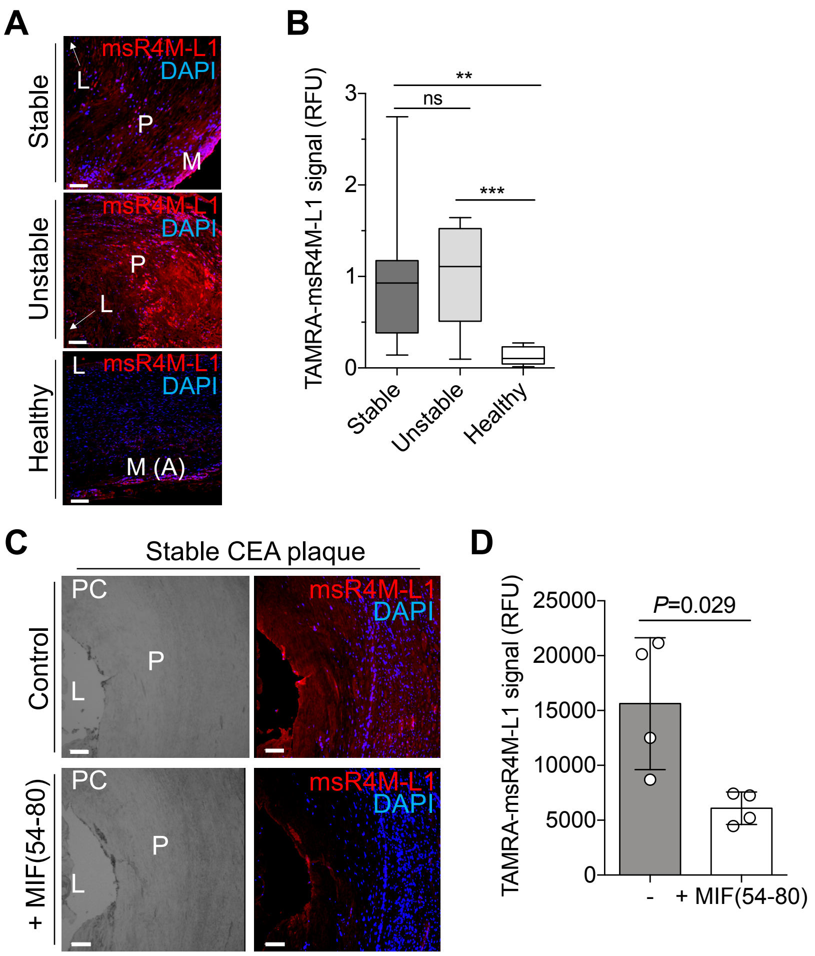


**Supplementary Figure 28 a-b** Quantification of the TAMRA-msR4M-L1 signal; **a** representative merged images after MIF staining with TAMRA-msR4M-L1 (red) and nuclear counterstain (DAPI, blue); **b** quantification of stainings according to **a** (as relative fluorescence units, RFU) from n=11 stable and n=12 unstable plaques, and n=9 healthy vessels. Vessel areas are indicated: P, plaque, L, lumen, M, media, A, adventitia. **c-d** MIF-specific staining of the TAMRA-msR4M-L1 probe is demonstrated by competition with MIF(54-80), a MIF peptide fragment that represents the MIF core binding region at the binding interface with CXCR4 ectodomain mimic msR4M-L1. **c** Representative images from a stable plaque from one CEA patient; left, phase contrast image (PC); right, merged image after MIF staining with TAMRA-msR4M-L1 (red) and nuclear counterstain (blue). Top panel (control), section was stained with 1.5 µM TAMRA-msR4M-L1; bottom panel (+MIF(54-80), same but co-incubation with 2 x molar excess of MIF(54-80). Red autofluorescence in control stainings without TAMRA-msR4M-L1 was negligible (not shown). Size bar is 100 µm. The section shown is representative for 5-8 fields-of-view from one section of one patient. **d** Quantification of stable plaque sections from four different CEA patients according to **c** (means ± SD; n = 4 independent CEA patients with means of n = 5-8 FoV each). Statistical analysis in **b** was performed by Kruskal-Wallis test with Dunn's multiple comparisons (** $P<0.01$, *** $P<0.001$, *ns*, not significant). Data in **b** are reported as box-whisker plot (stable (minimum: 0.1424, 25% percentile: 0.3845, center: 0.9279, 75% percentile: 1.174, maximum: 2.747; unstable (minimum: 0.09721, 25% percentile: 0.5124, center: 1.108, 75% percentile: 1.524, maximum: 1.644); healthy (minimum: 0.01442, 25% percentile: 0.4392, center: 0.1033, 75% percentile: 0.2309, maximum: 0.2749). Statistical analysis in **d** was performed by two-tailed Mann-Whitney test. Source data are provided as a Source Data file.

## Supplementary Tables

**Supplementary Table 1.** Summary list and molecular masses of all synthesized CXCR4 ectodomain peptides applied in this study.

| **Peptide acronym** | **Peptide sequence** | **[M+H]$^+$ theoretical**[a] | **[M+H]$^+$ experimental**[a] |
|---|---|---|---|
| **ECL1** | $NH_2$-DAVANWYFGNFLCK-$CONH_2$ | 1646.79 | 1670.02[b] |
| **ECL2** | $NH_2$-DRYICDRFYPNDLWV-$CONH_2$ | 1973.94 | 1974.38 |
| **msR4M-L1** | ECL1-6-Ahx-12-Ado-ECL2 | 3912.92 | 3913.66 |
| **msR4M-L2** | ECL1-O2Oc-12 Ado-ECL2 | 3944.93 | 3945.48 |
| **msR4M-L1ox** | ECL1-6-Ahx-12-Ado-ECL2<br>\| \|<br>$C^{109}$ — $C^{186}$ | 3910.90 | 3911.37 |
| **msR4M-L2ox** | ECL1-O2Oc-12-Ado-ECL2<br>\| \|<br>$C^{109}$ — $C^{186}$ | 3942.91 | 3943.47 |
| **msR4M-LS** | ECL1 ECL2<br>\| \|<br>$C^{109}$ — $C^{186}$ | 3618.68 | 3618.78 |
| **Fluos-ECL1** | Fluos-DAVANWYFGNFLCK-$CONH_2$ | 2004.75 | 2027.93[b] |
| **Fluos-ECL2** | Fluos-DRYICDRFYPNDLWV-$CONH_2$ | 2330.92 | 2332.08 |
| **Fluos-msR4M-L1** | Fluos-ECL1-6-Ahx-12-Ado-ECL2 | 4270.91 | 4271.72 |
| **Biotin-6-Ahx-msR4M-L1** | Biotin-6-Ahx-ECL1-6-Ahx-12 Ado-ECL2 | 4252.06 | 4252.47 |
| **TAMRA-msR4M-L1** | TAMRA-ECL1-6-Ahx-12-Ado-ECL2 | 4325.06 | 4363.81[c] |
| **msR4M-L1(2xAla)** | [$A^{104}$, $A^{107}$]-ECL1-6-Ahx-12-Ado-ECL2 | 3760.86 | 3761.37 |
| **Fluos-msR4M-L1(2xAla)** | Fluos-[$A^{104}$, $A^{107}$]-ECL1-6-Ahx-12-Ado-ECL2 | 4118.85 | 4119.66 |
| **msR4M-L1(7xAla)** | [$A^{102}$, $A^{103}$, $A^{104}$, $A^{107}$]-ECL1-6-Ahx-12-Ado-[$A^{189}$, $A^{190}$, $A^{195}$]-ECL2 | 3270.69 | 3271.11 |
| **Fluos-msR4M-L1(7xAla)** | [$A^{102}$, $A^{103}$, $A^{104}$, $A^{107}$]-ECL1-6-Ahx-12-Ado-[$A^{189}$, $A^{190}$, $A^{195}$]-ECL2 | 3628.68 | 3629.01 |
| **Fluos-msR4M-L2** | Fluos-ECL1-O2Oc-12 Ado-ECL2 | 4302.92 | 4303.28 |

| | | | |
|---|---|---|---|
| **Fluos-msR4M-L1ox** | Fluos-ECL1-6 Ahx-12 Ado-ECL2<br>$C^{109}$ ——— $C^{186}$ | 4268.89 | 4268.95 |
| **Fluos-msR4M-L2ox** | Fluos-ECL1-O2Oc-12 Ado-ECL2<br>$C^{109}$ ——— $C^{186}$ | 4300.90 | 4301.39 |
| **Fluos-msR4M-LS** | Fluos-ECL1 ECL2<br>$C^{109}$ ——— $C^{186}$ | 3976.67 | 3976.14 |

**Table legend.** The theoretical masses $[M+H]^+$ (**a**) were calculated based on the monoisotopic mass (M) and are indicated for all peptides compared to the $[M+H]^+$ (**a**), ($[M+Na]^+$ (**b**), or $[M+K]^+$ (**c**) masses determined experimentally by MALDI-MS. ECL, extracellular loop; msR4M, MIF-specific CXCR4 ectodomain mimic; Fluos, fluorescein; TAMRA, 5-carboxy-tetramethylrhodamine; 6-Ahx, 6-aminohexanoic acid; 12-Ado, 12-amino-dodecanoic acid; O2Oc, 8-amino-3,6-dioxaoctanoic acid; NH2-, indicates free N-terminus of respective peptide; -$CONH_2$, indicates C-terminal amidation of respective peptide; the numbering of the Cys residues refers to their position in the protein sequence of human CXCR4[3].

**Supplementary Table 2.** Binding affinities ($K_D$) between the CXCR4 ectodomain peptide msR4M-L1 and partial MIF peptides as determined by fluorescence titration spectroscopy.

| **Overall screen of MIF(2-115)** | | **Screen of binding region MIF(38-80)** | |
|---|---|---|---|
| MIF sequence | App. $K_D$ (nM) | MIF sequence | App. $K_D$ (nM) |
| 2-16 | >20000 | 38-80 | 57.1 ± 7.8 |
| 6-23 | >20000 | 38-60 | >20000 |
| 13-27 | >20000 | 38-64 | >20000 |
| 18-32 | >20000 | 38-68 | 696.3 ± 26.3 |
| 23-38 | >20000 | 38-72* | 160.7 ± 89.6 |
| 28-43 | >20000 | 38-76 | 42.2 ± 27.9 |
| 38-80 | 57.1 ± 7.8 | 50-60 | >5000 |
| 69-90 | >20000 | 50-80 | 55.2 ± 9.9 |
| 76-90 | >20000 | 51-67* | >20000 |
| 81-94 | >20000 | 54-80 | 70.6 ± 14.2 |
| 81-95 | 481.1 ± 43.5 | 55-80 | 479.4 ± 154.7 |
| 81-102 | 480.2 ± 83.1 | 56-69* | 1819 ± 491 |
| 82-95 | >10000 | 57-80 | 283.1 ± 57.7 |
| 86-100 | >20000 | 58-80 | 540.4 ± 206.3 |
| 91-105 | >10000 | 60-74* | >20000 |
| 101-115 | >20000 | 60-80 | 1758 ± 272 |
| | | 62-80 | >10000 |

**Table legend.** Fluorescence spectroscopic titrations were performed as described in **Fig. 2** of the main manuscript. Fluos-msR4M-L1 was generally applied at a concentration of 5 nM; the asterisk (*) denotes those peptide titrations for which Fluos-msR4M-L1 was used at 10 nM. The numbering of the sequence of human MIF (2-115) refers to the cDNA sequence and accounts for the notion that the N-terminal Met-1 residue is processed [4, 5]. MIF, human macrophage migration-inhibitory factor; app., apparent. The source data for MIF peptides 6-23, 38-80, 54-80, and 62-80 are provided within the Source Data files for Fig. 2.

**Supplementary Table 3.** Therapeutic treatment of atherogenic *Apoe*$^{-/-}$ mice with msR4M-L1 does not affect blood leukocytes and lipid levels. Blood cell count, body weight and serum lipid levels from mice *Apoe*$^{-/-}$ mice on cholesterol-rich high-fat diet (HFD) for 4.5 weeks and treated with msR4M-L1 or vehicle control.

| | Vehicle | msR4M-L1 | *P* value |
|---|---|---|---|
| **Serum lipid levels** | | | |
| Cholesterol (mg/dL) | 693.4 ± 25.2 | 647.4 ± 19.2 | 0.1686 |
| Triglycerides (mg/dL) | 161.3 ± 5.7 | 156.7 ± 3.8 | 0.5203 |
| **Blood cell counts** | | | |
| Leukocytes (per µL) | 4874 ± 673 | 4632 ± 286 | 0.7490 |
| Monocytes (per µL) | 702 ± 128 | 552 ± 51 | 0.3096 |
| Lymphocytes (per µL) | 3070 ± 560 | 2347 ± 50 | 0.2343 |
| Neutrophils (per µL) | 2115 ± 272 | 1897 ± 46 | 0.4538 |
| **Body weight** | | | |
| Weight (g) | 22.8 ± 0.4 | 22.3 ± 0.2 | 0.2848 |

**Table legend.** Shown are means ± SD. *P*-values calculated by Student's t-test. N=5 (blood cell counts), n=7 (body weight), and n=11 (lipids) per group. Source data are provided as a Source Data file.

**Supplementary Table 4.** Characteristics of the atherosclerotic patients undergoing carotid endarterectomy (CEA).

| Patient characteristics | Staining with TAMRA-msR4M-L1 | | | MIF gene expression (qPCR) | | |
|---|---|---|---|---|---|---|
| | Stable (n=11) | Unstable (n=12) | *P* value[2] | Stable (n=19) | Unstable (n=20) | *P* value[2] |
| Age (y)[1] | 65.5±2.2 | 71.9±3.2 | 0.391 | 66.6±1.7 | 68.5±2.2 | 0.493 |
| Sex (male, %) | 41.7 | 61.5 | 0.341 | 57.9 | 80.0 | 0.142 |
| Neurological symptoms (%) | 36.4 | 25.0 | 0.575 | 15.8 | 40.0 | 0.098 |
| Hypertension (%) | 72.7 | 83.3 | 0.559 | 68.4 | 80.0 | 0.421 |
| Diabetes mellitus (%) | 36.4 | 8.3 | 0.113 | 36.8 | 35.0 | 0.908 |
| Hyperlipidemia (%) | 63.6 | 66.7 | 0.886 | 73.7 | 60.0 | 0.378 |
| Smoking (%) | 54.6 | 50.0 | 0.837 | 52.6 | 35.0 | 0.279 |
| CKD[1] (%) | 0 | 16.7 | 0.171 | 10.5 | 5.0 | 0.530 |
| Coronary heart disease (%) | 9.1 | 25.0 | 0.337 | 21.1 | 10.0 | 0.352 |
| PAD (%) | 0 | 8.3 | 0.350 | 5.3 | 20.0 | 0.178 |
| Aspirin/Clopidogrel (%) | 100.0 | 91.7 | 0.350 | 94.7 | 90.0 | 0.591 |
| Beta-blocker (%) | 27.3 | 25.0 | 0.907 | 31.6 | 40.0 | 0.595 |
| ACE-inhibitors[3] (%) | 18.2 | 33.3 | 0.432 | 21.1 | 30.0 | 0.535 |
| Statins (%) | 90.0 | 75.0 | 0.337 | 79.0 | 85.0 | 0.633 |
| Diuretics (%) | 9.1 | 16.7 | 0.610 | 0 | 25.0 | 0.019 |

**Table legend.** All atherosclerotic carotid tissue samples used for analysis showed an advanced stage of atherosclerosis (types V–VII according to the American Heart Association (AHA) guidelines). [1] Age of healthy controls (TAMRA-msR4M-L1 cohort: 57.4 ± 1.9 years; mRNA cohort: 56.8 ± 3.5 years). [2] *P* values refer to stable *versus* unstable samples (unpaired two-tailed T-test). Abbreviations: CKD, chronic kidney disease; ACE, angio-tensin-converting enzyme; PAD, peripheral artery disease.

## References cited in Supplementary Part


1. Qin, L. *et al.* Structural biology. Crystal structure of the chemokine receptor CXCR4 in complex with a viral chemokine. *Science* **347**, 1117-1122 (2015).
2. Rajasekaran, D. *et al.* Macrophage migration inhibitory factor-CXCR4 receptor interactions: evidence for partial allosteric agonism in comparison with CXCL12 chemokine. *J Biol Chem* **291**, 15881-15895 (2016).
3. Wu, B. *et al.* Structures of the CXCR4 chemokine GPCR with small-molecule and cyclic peptide antagonists. *Science* **330**, 1066-1071 (2010).
4. Bernhagen, J. *et al.* Purification, bioactivity, and secondary structure analysis of mouse and human macrophage migration Inhibitory factor (MIF). *Biochemistry* **33**, 14144-14155 (1994).
5. Weiser, W.Y. *et al.* Molecular cloning of cDNA encoding a human macrophage migration inhibtion factor. *Proc Natl Acad Sci USA* **86**, 7522-7526 (1989).
