# Supplementary material for: Designed CXCR4 mimic acts as a soluble chemokine receptor that blocks atherogenic inflammation by agonist-specific targeting
Source: Nat Commun. 2020 Nov 25;11:5981. doi: 10.1038/s41467-020-19764-z (PMC7689490; doi:10.1038/s41467-020-19764-z)
Supplement: Supplementary file 3 — Description of Additional Supplementary Files [file 41467_2020_19764_MOESM3_ESM.pdf]

Description of Additional Supplementary Files

File Name: Supplementary Movie 1

Description: msR4M-L1 inhibits leukocyte adhesion under physiological flow in whole-mount carotid arteries prepared from atherosclerotic mice Apoe$^{-/-}$ mice on Western-style HFD. The video represents a z sectioning video scan (single field of view) through a whole-mount carotid artery using multiphoton laser-scanning microscopy (MPM). msR4M-L1 (red) versus vehicle control (green) msR4M-L1 was in vivo-injected on the three days before vessel preparation and perfused leukocytes stained accordingly.
